# Fast magnetic resonance spectroscopic imaging techniques in human brain- applications in multiple sclerosis

**DOI:** 10.1186/s12929-017-0323-2

**Published:** 2017-02-28

**Authors:** Oun Al-iedani, Jeannette Lechner-Scott, Karen Ribbons, Saadallah Ramadan

**Affiliations:** 1 School of Health Sciences, Faculty of Health and Medicine, University of Newcastle, Callaghan, NSW 2308 Australia; 2 School of Medicine and Public Health, Faculty of Health and Medicine, University of Newcastle, Callaghan, NSW 2308 Australia; 3 Department of Neurology, John Hunter Hospital, Lookout Road, New Lambton, NSW 2305 Australia; 4 Hunter Medical Research Institute, Kookaburra Circuit, New Lambton, NSW 2305 Australia

**Keywords:** Fast MRSI, Spiral, EPSI, Human, In vivo, Multiple Sclerosis

## Abstract

Multi voxel magnetic resonance spectroscopic imaging (MRSI) is an important imaging tool that combines imaging and spectroscopic techniques. MRSI of the human brain has been beneficially applied to different clinical applications in neurology, particularly in neurooncology but also in multiple sclerosis, stroke and epilepsy. However, a major challenge in conventional MRSI is the longer acquisition time required for adequate signal to be collected. Fast MRSI of the brain in vivo is an alternative approach to reduce scanning time and make MRSI more clinically suitable.

Fast MRSI can be categorised into spiral, echo-planar, parallel and turbo imaging techniques, each with its own strengths. After a brief introduction on the basics of non-invasive examination (^1^H-MRS) and localization techniques principles, different fast MRSI techniques will be discussed from their initial development to the recent innovations with particular emphasis on their capacity to record neurochemical changes in the brain in a variety of pathologies.

The clinical applications of whole brain fast spectroscopic techniques, can assist in the assessment of neurochemical changes in the human brain and help in understanding the roles they play in disease. To give a good example of the utilities of these techniques in clinical context, MRSI application in multiple sclerosis was chosen. The available up to date and relevant literature is discussed and an outline of future research is presented.

## Background

### MRS and MRSI

Magnetic resonance spectroscopy (MRS) is a technique used to identify and quantify metabolites in vivo, giving chemical and quantitative information rather than anatomical information, as in routine MR imaging. MRS interrogates a three dimensional volume of tissue within the body positioned in a MR scanner, to produce a “spectrum” of information about existing chemicals and their relative concentrations. Most applications and technical developments of MRS have focused on the human brain, including clinical studies and increased understanding of the pathology of Parkinson’s disease [1], Alzheimer’s disease [2], stroke [3] and multiple sclerosis (MS) [4, 5]. MR spectra can be acquired from many chemical elements. However, proton (^1^H) spectroscopy provides a large sensitivity advantage over other nuclei used in MRS (e.g. ^31^P and ^13^C). This is because it has the greatest gyromagnetic ratio (γ) of non-radioactive nuclei and a high natural abundance. This sensitivity is augmented compared to other nuclei, due to propitious metabolite relaxation times and because several essential brain metabolites have multiple protons.

In 1985, Bottomley et al., used a slice-selective spin-echo excitation and frequency-selected water suppression (at 1.5 tesla (T)) to obtain the first spatially localised human brain spectrum, at a time when spatial localisation and spectral resolution were limited [6]. Many spatial localisation techniques were developed in the 1980s, when the technology was in its elementary stages and faced many difficulties in implementation and efficiency. Presently, the two most basic and common techniques used in spectroscopy are Stimulate Echo Acquisition Mode (STEAM) [7, 8] and Point RESolved Spectroscopy (PRESS) [9, 10] which are based on three slice-selective pulses applied in orthogonal planes.

MRSI can also be used in an MR scanner to fully cover an organ, e.g. brain, by giving a spectroscopic signature from each part of this organ. It is a method used to collect spectroscopic data and spatial distribution of metabolites using multiple-voxel locations within a single measurement. Multi-voxel spectroscopy (2 or 3 dimensional (2D or 3D)) plays a particularly prominent role, not only in increasing the spatial coverage, but also in improving the efficiency of data collection. Major disadvantages of the technique are long acquisition times, lack of adequate signal-to-noise ratio (SNR), insufficient water and lipid suppression and limited spatial coverage; these elements pose major constraints and limitations. Despite these disadvantages, MRSI has the potential to play a significant role to assist in clinical diagnosis and treatment planning. Many different MRSI acquisition methods have been developed, including conventional and fast MRSI methods, each of which has its own advantages and disadvantages.

MRSI was initially conceptually proposed and implemented in a phantom with varying phosphorus chemical shift composition by Brown et al. in 1982 [11] . The method used a sequence of radio-frequency (RF) and magnetic field gradient pulses to measure chemical shift distribution across a rectangular grid. Simple Fourier transformation was applied to recover the original chemical shift distribution. The first in vivo application was carried out on a human forearm on a 1.5 T magnet by Pykett et al. [12].

### Multiple sclerosis (MS)

MS is an immune-mediated neuronal disorder in which inflammatory cells attack the myelin of the central nervous system (CNS), leading to varying extents of neuroaxonal injury, demyelination and gliosis by affecting both the brain and spinal cord [13, 14]. Typically, symptoms of MS are based on the location of the plaque and most patients experience initially exacerbations and remissions due to inflammation and recovery with remyelination which, in the later stages, is exhausted and then leads to persistent symptoms. Clinically, MS can be classified into: (a) relapsing remitting MS (RRMS) that accounts for 85% of MS patients, and is characterised with remission phases (stability) and relapse or exacerbation [15], (b) chronic progressive MS is divided into primary progressive MS (PPMS), secondary progressive MS (SPMS) and progressive relapsing (PRMS). However, the new classification by Lublin [16] aims to characterise progressive disease according to its clinical and MRI activity. PPMS is defined by slowly progressing disability from onset, characterised by localised subpial inflammation without blood brain barrier disruption [17]. The diagnosis and management of MS is increasingly reliant on non-invasive MR modalities. Indeed, the current diagnostic criteria for MS [18] includes specific MR imaging features which provides evidence of dissemination in space and time of brain and spinal cord lesions. Recent guidelines regarding the frequency of MRI protocols and frequency of MR evaluations [19] suggest MR imaging be undertaken between every 6 months and 2 years for all RRMS patients, to monitor new and enhancing lesions and contribute to the medical management of the relapsing form of the disease. However, in contrast, there are no current reliable markers to evaluate therapeutic efficacy in the progressive forms of MS, which has been a major obstacle in the development of new disease-modifying therapies.

^1^H-MRS might add to the specificity of diagnosis and clinical management by the potential identification of new disease biomarkers [20, 21]. ^1^H-MRS provides a unique potential to evaluate biochemical alteration in MS. In light of this, neurochemical changes of the brain are related to the metabolite concentration levels. For instance, a reduction of N-acetylaspartate (NAA) level, which is an amino acid derivative and has a high concentration in the brain, reflects axonal degeneration or loss, [20, 22] while increased Creatine (Cr) levels, known to play an important role in cellular energy metabolism, can be indicative of gliosis in MS patients [23]. Furthermore, increased resonance intensity of Choline (Cho) indicates an altered turnover of cell membranes level in steady state, and finally, alteration in myo-inositol (mI) concentration can indicate increased glial cell activity or changes in the inflammatory cells [24].

While existing MR protocols used in MS focus on changes in white matter lesions it is evident that there is a disparity between lesion load and clinical disability [25] and current MR protocols have limited sensitivity in detecting changes in gray matter. This leaves neuroradiologists with the dilemma on how to best accurately evaluate pathological changes occurring across the entire MS brain [26].

Several reports have primarily studied MS patients using single-voxel spectroscopy (SVS) methods to evaluate spectroscopic changes of brain metabolites and their ratios in several ROI including normal appearing white matter (NAWM) and (gray matter) GM [27, 28], in addition to whole brain NAA (WBNAA) [29] at different fields strengths (1.5–3 T) and echo time (TE) values (20–70 ms) [30]. However, these techniques have successfully collected data from a limited region of the brain, within acceptable acquisition times, the real challenge for these methods is to be able to perform a metabolite mapping covering the whole brain, with high spatial resolution and short TE in order to estimate neurochemical changes within larger brain regions in one session. The potential usefulness for such techniques in a clinical setting is also dependent on the acquisition time for the MRS or MRSI profile. If the intention is to run these novel MR metrics in parallel to the standard MS MR protocols, acquisition times and quantification procedures need to be optimised to make this application feasible.

### Scope of the review

In this article, the underlying principles of MRS will be described and the different MRSI techniques compared, focussing on recent advances in high-speed MRSI methods. MS will be used as an example pathology in a clinical setting where MRSI techniques are being applied to map brain metabolic changes in different areas of the brain and at different disease stages to evaluate the potential use of the technique as a tool in disease diagnosis and clinical management.

### Data acquisition techniques

#### Single-voxel techniques

**Fig. 1:**
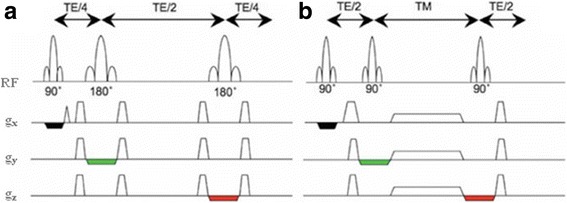
Two single-voxel localisation methods: **a** the PRESS sequence; **b** the STEAM sequence. Note that the three orthogonal slice-selective gradient pulses are indicated by *black*, *green* and *red* colours in the schematic representation. Reproduced with permission from [39]

In general, the spatial coverage of MRS falls into two categories, either localised SVS or multi-voxel MRSI [31]. The performance of these techniques is based on a slice-selective excitation of RF pulses in variant forms, combined with magnetic-field gradients. The primary principle of SVS is that it sequentially excites three orthogonal slices, whose intersection defines the volume of interest (VOI). Then the generated echo signal is accumulated so that only the signal from the voxel, where all three slices intersect, survives. To ensure signal fidelity, signals from outside the VOI can be eliminated by dephasing crusher gradients and phase cycling of RF pulses [32]. In SVS techniques, STEAM or PRESS are typically used to excite the VOI within the brain as a standard method of clinical imaging [33]. Figure 1 shows that single-voxel localisation methods collect signals from a rectangular region of interest (ROI). PRESS (Fig. 1a) uses a double echo technique; where the procedure consists of an initial 90° RF pulse applied with an x-gradient to excite a slice followed by second and third 180° RF pulses applied with two other gradient pulses along y and z planes, respectively. Also, appropriate spoiler gradients along all gradient channels are used to dephase undesired coherence. In STEAM (Fig. 1b), however, three 90° RF pulses are used in order to obtain the stimulated echo. Accompanying this operation, a large spoiler gradient pulse should be employed to dephase other created signals during the mixing time (TM). A second 90° RF pulse is applied after half of TE from the first 90° RF pulse. In order to eliminate any undesired signals, spoiler gradients need to be carefully applied during TE on all gradient channels.

**Fig. 2:**
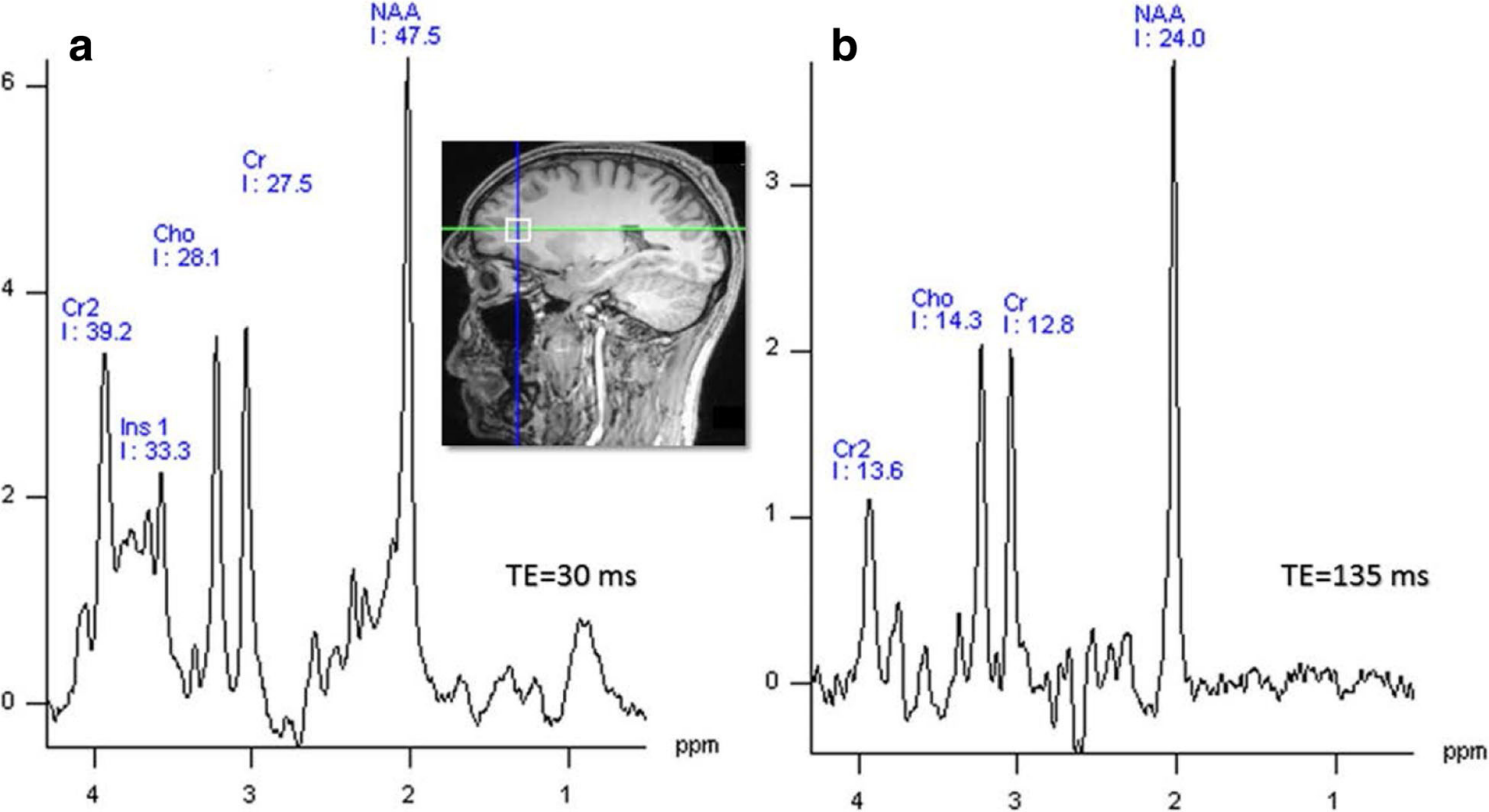
Signal obtained from prefrontal cortex (PFC) of voxel size (1.5 cm^3^) from a healthy subject: **a** at short TE and **b** at long TE using PRESS approach on a 3 T scanner (Prisma, Siemens, Erlangen)

To determine which sequence is to be selected is largely dependent on the specific metabolites to be detected in the study. STEAM uses symmetric RF pulses and optimised gradient waveforms to minimise TE, so it is applicable to instances that require short TE values for the retention of metabolites with short T2. PRESS accommodates the requirements for studies that have a preference for longer TE and it comes with higher signal yield due to the 180° RF pulses used [8, 34]. PRESS can still be used in cases where T2 is long and T2* (T2 with static magnetic field (Bo) inhomogeneity contributions) is short. Figure 2 shows typical single-voxel spectra acquired on a 3 T Prisma scanner (Siemens, Erlangen) at different TE value.

#### Conventional multi-voxel techniques

Single-voxel techniques are invariably used in clinical settings, however, SVS techniques are restricted by their limited coverage and coarse spatial resolution. These constraints can be overcome by MRSI techniques [11]. For more global coverage, MRSI can also be extended to 3D-MRSI [35–37].

**Fig. 3:**
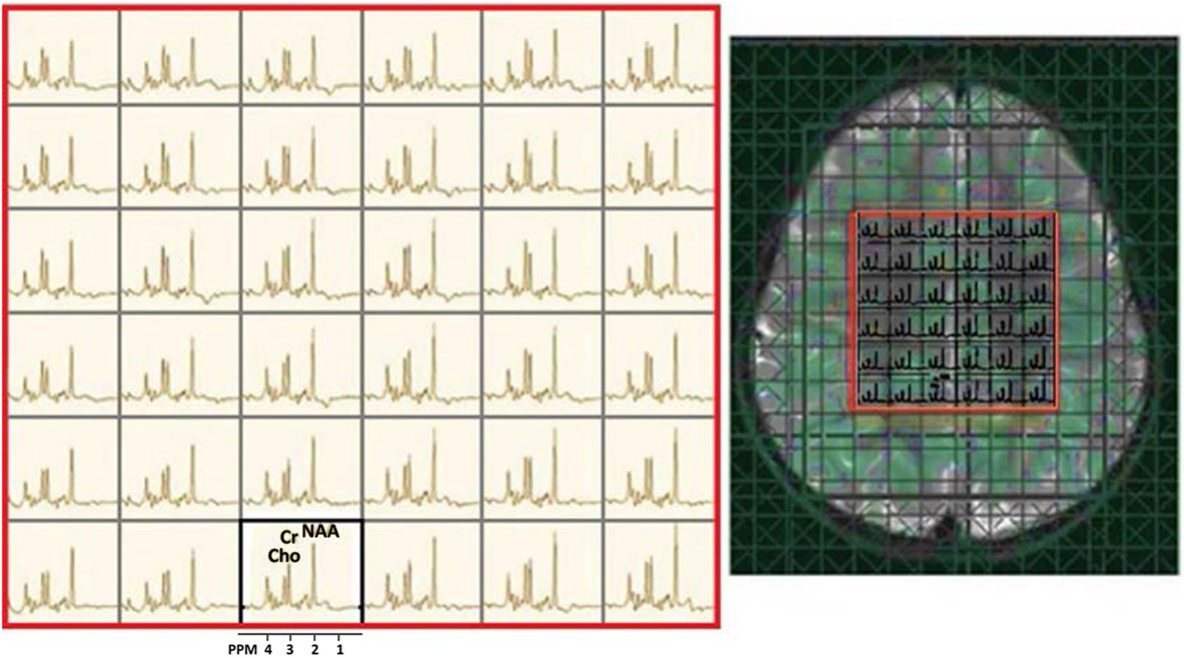
MRSI data acquired from a 3-year-old girl with an idiopathic developmental delay. Data was acquired using a 2D PRESS-MRSI at 3 T (TE: 135 ms) in the *axial plane* with voxel size of 1.5 cm^3^. Reproduced with permission from [39]

The conventional 2D- and 3D-MRSI studies of the human brain, which are usually based on PRESS sequence, have numerous challenges which include long acquisition times, low SNR and extra-voxel contamination. Scan time is proportional to number of phase-encoding steps, repetition time (TR) and number of averages [8, 38, 39]. Although PRESS-MRSI was designed for routine scanners, the scan times were still too long for clinical applications especially in 3D mode [40]. In addition to long scanning time, the homogeneity of magnetic field becomes an important issue especially when PRESS-MRSI is used to map whole brain. For the latter issue, for example, higher order shimming was developed to improve the field homogeneity for larger volumes [41]. Other MRSI issues have been expanded upon elsewhere [38]. Figure 3 shows an example of 2D PRESS-MRSI data at 3 T [39].

To overcome the above challenges, fast MRSI techniques were introduced as an improved alternative to facilitate implementation of this technique in the clinic, and to eliminate challenges associated with conventional MRSI techniques.

#### Parallel imaging

Parallel imaging techniques, such as sensitivity encoding (SENSE) [42], simultaneous acquisition of spatial harmonics (SMASH) [43] and generalised auto-calibrating partial parallel acquisition (GRAPPA) [44], have been introduced and commonly used to accelerate MRI techniques and can also be applied to improve the temporal performance of conventional MRSI [45–47]. In parallel imaging, signal sensitivity and spatial encoding can be improved by using multiple receiver coils, whereby the number of needed k-space lines decreases with considerable acceleration in the image acquisition.

**Fig. 4:**
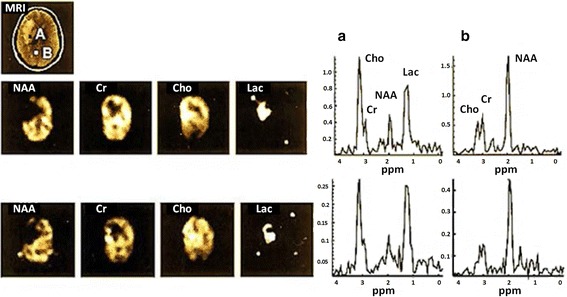
Illustrates the data spectroscopy and mapping of brain metabolite of conventional MRSI methods (*top line*) compared with SENSE-MRSI acquisition methods (*bottom line*) of **a** voxel in tumorous tissue and **b** healthy tissue; with an acquisition time of (14.02 min) and (3.37 min) respectively, and acquisition data parameter (TE/TR: 228/1500 ms), *slice thickness* (20 mm) and FOV (220 mm). Reproduced with permission from [50]

For SENSE-MRSI, the principal balance between acceleration of spatial encoding and noise amplification is an essential requirement due to two factors; the reduced number of phase-encoding steps, and the acceleration factor (R). It has been proposed that low SNR can be improved in parallel imaging based techniques by increasing the number of coil elements [48]. For example, the performance of SENSE based 2D-MRSI can be improved using an 8–12 channel-coil [47], and SENSE based 3D-MRSI using a 32 channel coils [49]. An important additional advantage of parallel imaging techniques is their compatibility with fast MRSI approaches discussed below. Figure 4 shows an example of a SENSE-MRSI data with an acquisition time of only 3.37 min [50].

#### Fast multi-voxel techniques

In order to study the whole brain, there must be a decrease in the scanning time and motion sensitivity. MRSI methods can be accelerated using time-varying gradients during the readout of spectroscopic imaging data [51–54]. Efficient spatial and spectral k-space sampling with time-varying gradients is a mechanism that can be used to address time limitations. The majority of k-space trajectories that are widely used in spectroscopic imaging are echo-planar and spiral trajectories [55–57]. Recent developments in the gradients hardware design made it possible to traverse the k-space within a shorter period of time within each repetition [58]. For this reason, spiral imaging has shown to be useful in specific applications such as cardiovascular and functional brain imaging applications [59].

A number of fast MRSI acquisition techniques designed to collect k-space data in three spatial dimensions have been reviewed elsewhere [48, 60]. Their main aim is not only to reduce acquisition time but also to minimise voxel signal contamination and improve metabolite mapping of the whole brain [61].

Many different strategies for fast MRSI have been used to gain high spatial resolution and to improve the time efficiency of MRSI experiments. The most common and effective of these approaches applied to the human brain are briefly described in this article.

### Spiral MRSI

**Fig. 5:**
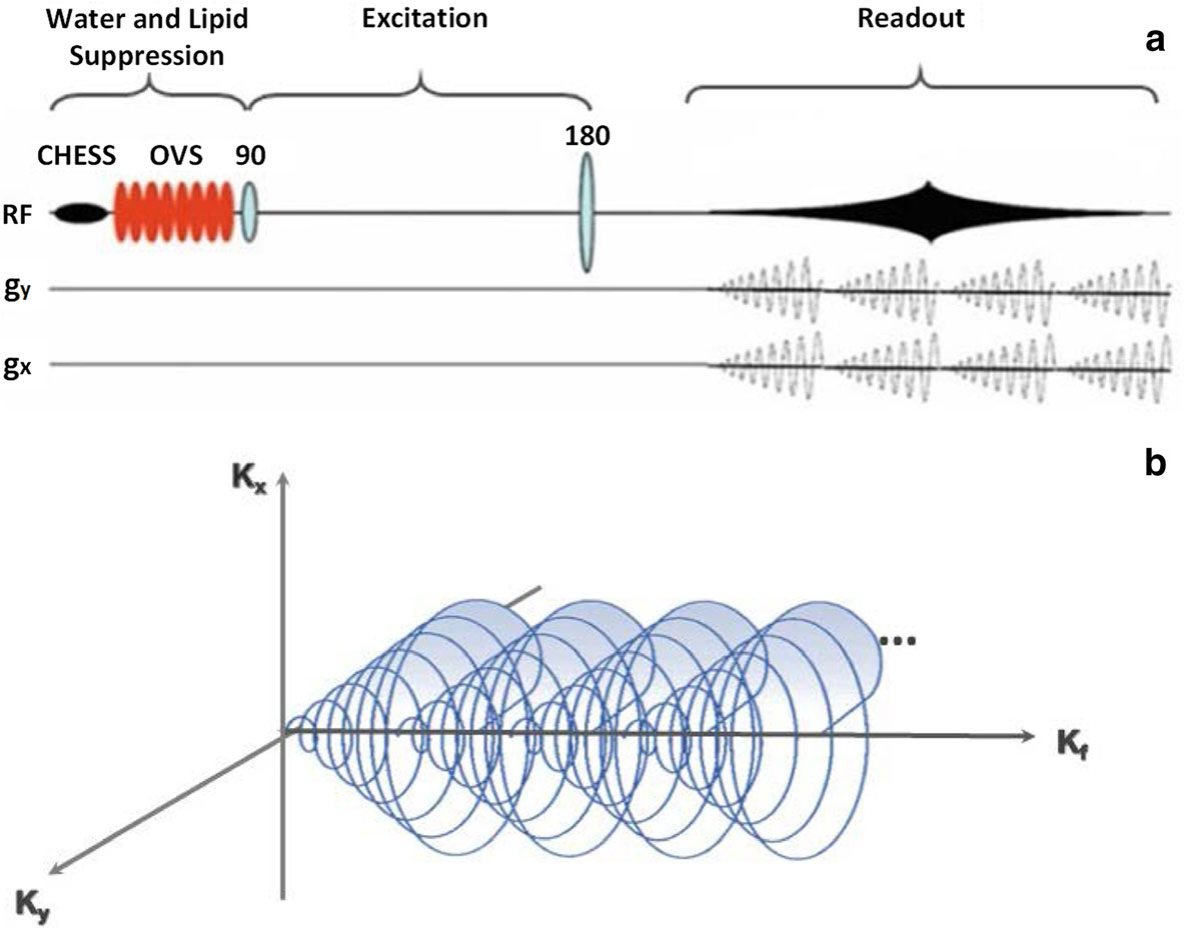
**a** In a spiral MRSI, two time-varying readout gradients are administered in the data acquisition period with oscillating spiral trajectories. **b** Outlines the projection of a k-space trajectory along the k_f_ axis. The spiral trajectories originate from the (k_x_, k_y_) plane and repeatedly run a path through the k_x_, k_y_, k_f_ spaces with multiple and simultaneous spiral trajectories increasing volumetric acquisition around the k_f_ axis. Reproduced with permission from [39, 56]

Spiral MRSI is a fast spectroscopic imaging technique that traverses k-space by spiral trajectories. Oscillating readout gradients are applied in a spectroscopic imaging sequence in two spatial dimensions during the data acquisition. These gradient waveforms (G_x_, G_y_) rapidly traverse spiral trajectories in two directions of k-space (k_x_, k_y_). These trajectories can be fully or partially covered within one TR as shown in Fig. 5.

Due to this ability, a sequence with spiral trajectory has a much quicker acquisition time compared to conventional MRSI methods [58]. This single-shot spiral-imaging technique sets a remarkable new standard for fast spectroscopic imaging.

Spiral MRSI was originally introduced by Adalsteinsson et al. [56] to evaluate the neurochemical change of metabolites in GM in patients with SPMS [4]. However, this technique has limitations in certain clinical applications (i.e. increased blurring and hardware limitation), and thus never became a widely used tool despite its advantages.

Data sampled in spiral spectroscopic imaging sequences are usually non-uniform, and thus acquired data has to be re-gridded to reconstruct the data onto a Cartesian k-space, where Fourier transformation can be applied [57, 59]. Due to the data collection being completely symmetric and sampled around the centre of k-space, several artefacts that are influenced by external variables such as motion or other instabilities are reduced [62]. As a result spiral MRSI offers shorter imaging time, higher spatial resolution, improved point spread function (PSF) and SNR.

Spiral spectroscopic imaging can be readily and effectively combined with other imaging-based techniques such as parallel imaging methods leading to Mayer et al. proposing their accelerated version of this technique for human brain at 3 T [63].

**Fig. 6:**
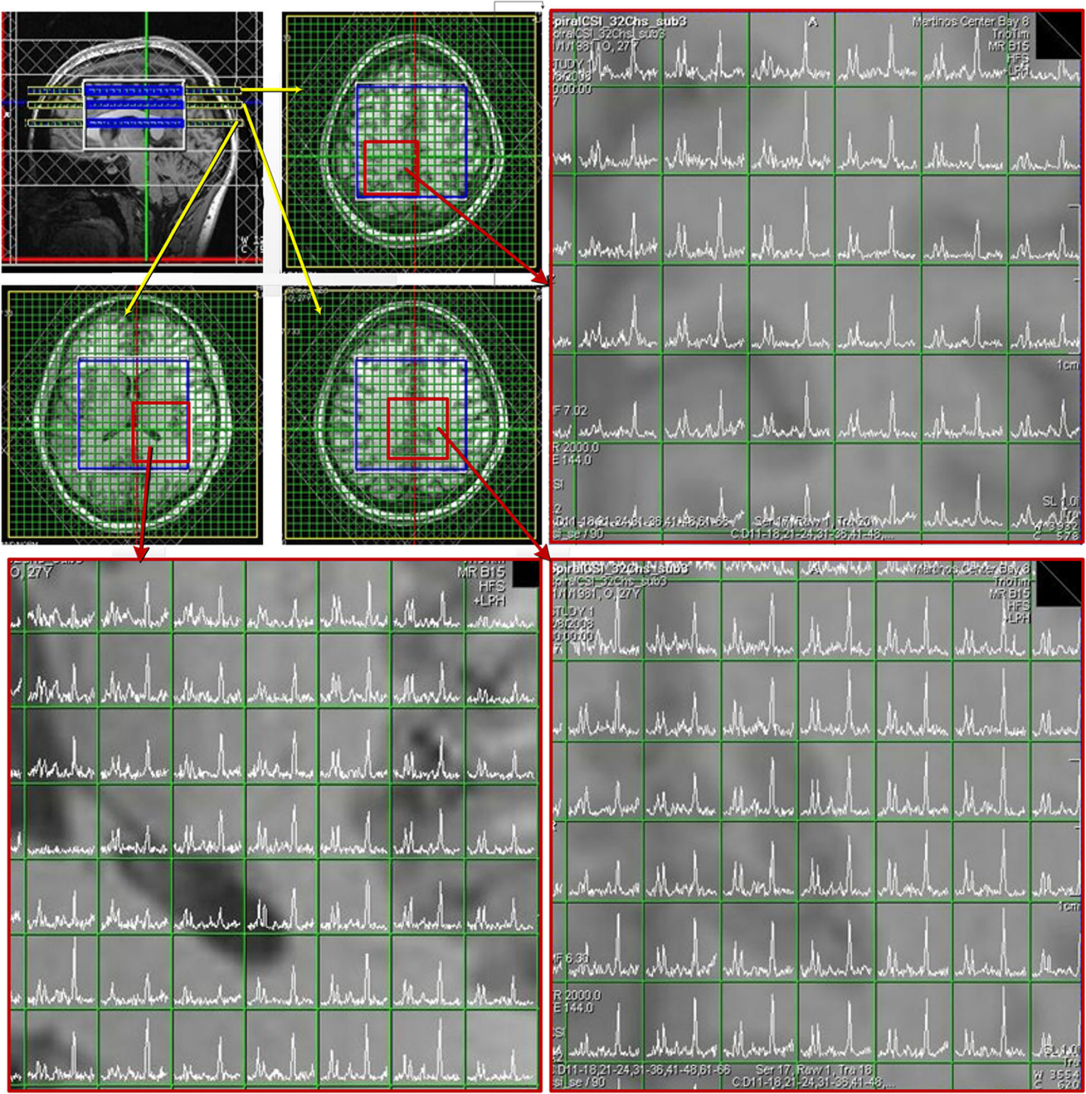
Displays the spectral data from three slices using a spiral MRSI technique at 3 T (TE/TR: 144 ms/2 s, FOV: 8 × 9 × 6 cm) using a 32-channel phased array head coil. Reproduced with permission from [58]

Recent work focussed on improving localisation and spectral quality of spiral MRSI [64–66]. These developments will have significant clinical impact on the study of human brain. Despite spiral MRSI having several ‘theoretical’ benefits, its major drawback is the high strain on gradient hardware as a result of its demanding trajectory design [58]. An example of the clinical application of the spiral MRSI at 3 T, with a data-acquisition time of 13.5 min, is shown in Fig. 6.

SENSE-based spiral MRSI [63] has been applied to address the challenges associated with their clinical application, e.g. volumetric coverage and evaluation of the neurochemical change of the whole human brain [67].

### Turbo spectroscopic imaging (turbo-MRSI)

**Fig. 7:**
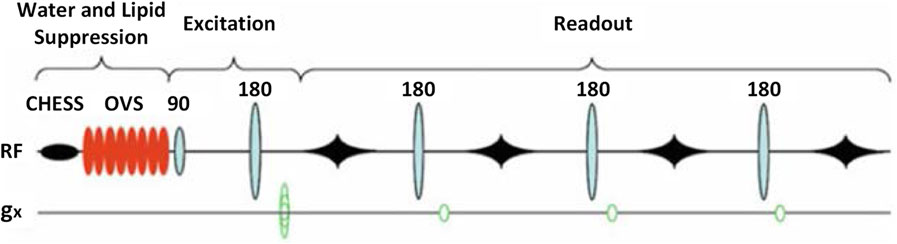
Readout strategy for Turbo-MRSI sequence using spin-echo imaging per excitation preceded by water and lipid suppression (CHESS and outer-volume suppression (OVS)). Reproduced with permission from [39]

MRSI can also be accelerated by multiple-echo refocussing which is analogous to turbo-spin-echo imaging as seen in Fig. 7. Determining the efficiency of this data collection strategy is largely dependent on rapid acquisition time and spatial resolution without signal loss of brain metabolites [68]. Turbo-MRSI techniques have proven successful in the past in detecting major brain metabolites such as NAA, Cho and Cr within an acceptable acquisition duration at 1.5 T [69]. Stengel et al. has succeeded in reducing the acquisition time to 6 min by using turbo-MRSI with four phase encodes per TR to study stroke patients [70].

Even though turbo-MRSI techniques have successfully mapped and assessed uncoupled brain metabolite distributions with long TE, mapping of coupled resonance metabolites (e.g. glutamine + glutamate (Glx)) proved to be a challenge. Fortunately, Yahya et al. [71] proposed modifications that allow the quantitation of Glx at TE of 100 ms and 170 ms in addition to halving acquisition time.

Turbo-MRSI can be combined with parallel imaging techniques such as SENSE to improve acquisition rate to obtain higher resolution (high sensitivity). Dydak et al. was able to design a turbo-SENSE-MRSI sequence that uses an echo train length of four to acquire spectroscopic data within two to three minutes and reduced acquisition times by about eight folds compared to conventional MRSI techniques [50].

Due to combining multiple-echo MRSI methods with parallel imaging techniques, high spatial resolutions MRSI become clinically feasible. Many challenging clinical applications have been achieved through the use of the turbo-SENSE-MRSI technique [72] involving high spatial encoding train (i.e. long multiple-echoes train) which only becomes feasible at 3 T. For instance, acquisition times are significantly reduced (~1 min) to obtain brain metabolites ratios Cho/NAA and Cr/NAA with a TE of 144 ms, even though SNR is reduced because of the longer echo train. In addition to these clinical successes, turbo-MRSI techniques [73] have made it possible to evaluate brain metabolite levels within the pons, accumulating spectroscopic data within very short periods of time (1 min 20 s) using long TE (288 ms) at 1.5 T.

The advent of the turbo-MRSI technique has made faster data acquisition possible, although with a major drawback of lowering spectral resolution, due to the short time between consecutive refocusing pulses [70, 73]. The second disadvantage is the drop in SNR as a consequence of the increase in spatial encoding trains of more than two, as the spatial encoding maintains a balance between the output of acquisition scan time and SNR [50, 72].

### Echo-planar spectroscopic imaging (EPSI)

The introduction of echo-planar imaging (EPI) originally proposed by Mansfield [74] has facilitated the development of EPSI on conventional clinical MRI scanners. The latter technique made the mapping of spatial metabolite distributions in the brain possible, accelerating spectral data acquisition compared to conventional MRSI, therefore creating an exceptionally fast imaging technique. New improvements to the readout frame of EPI techniques meant that an oscillating readout gradient can be reproducibly used in EPSI. EPSI encoding method that uses multiple-slice or PRESS excitation in 2D or 3D-MRSI [75, 76] became the method of choice. These improvements led to the advent of EPSI to change how MRSI is applied in a clinical setting.

**Fig. 8:**
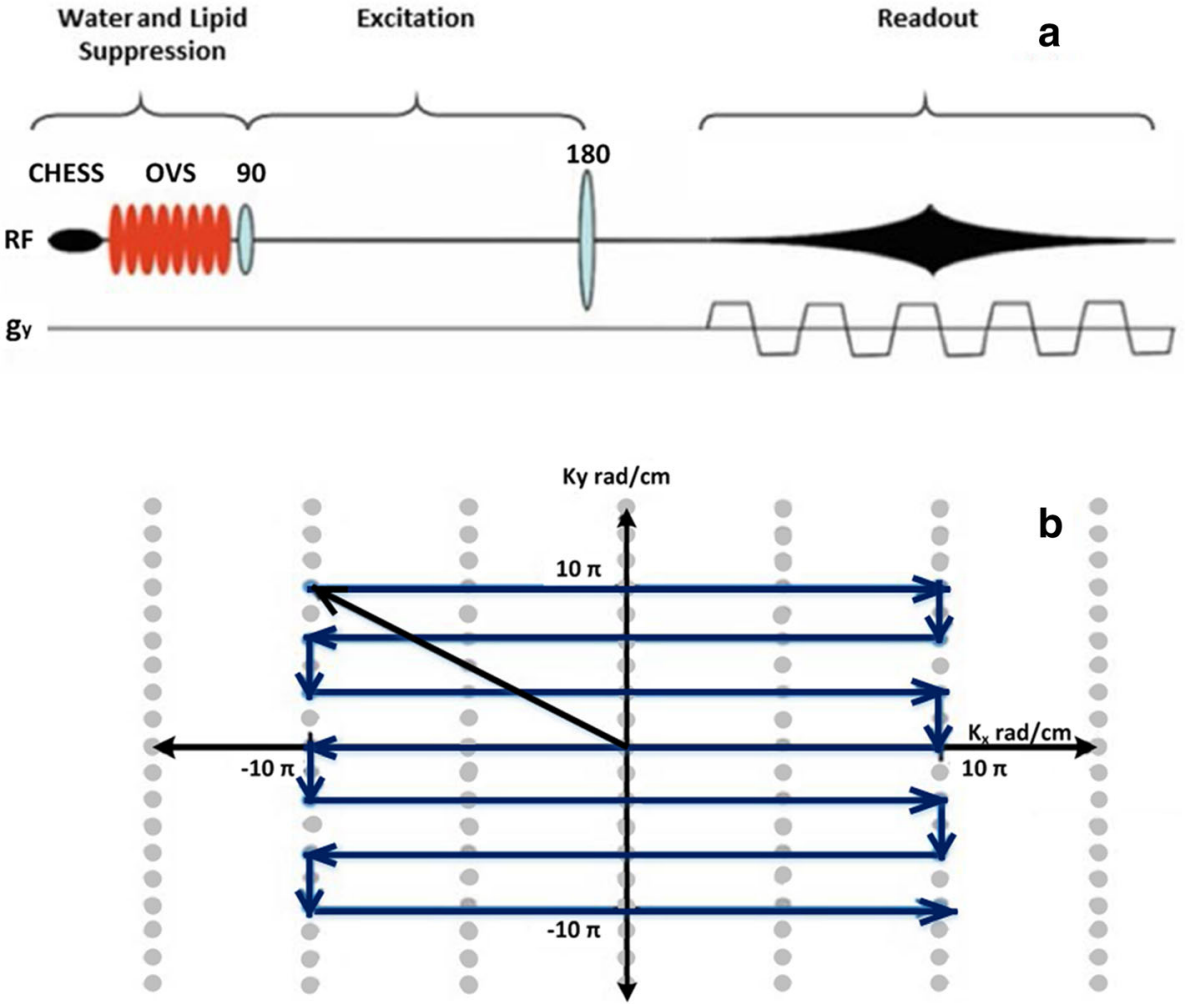
**a** EPSI sequences are applied to encode localised spectra with a single readout gradient. **b** k-space trajectories of echo-planar spectroscopic imaging indicate data acquisition in one TR of the pulse sequence during spectral encoding. Reproduced with permission from [39]

In the last decade, EPSI were widely used to acquire MRSI data in a shorter scanning time by encoding spatial and spectral dimensions in a single readout gradient (Fig. 8a). This fact is based on rapid k-space sampling per excitation that allows planar data collection on rectilinear trajectories (Fig. 8b).

Echo-planar encoding has proved particularly useful in ^1^H-MRSI applications. Its application has improved performance in covering large volumes due to its improved spatial and temporal resolution, compared to typical conventional phase-encoded MRSI.

The spectroscopic images for distribution of the major metabolites in the human brain were first obtained with 3D-EPSI technique by Posse et al. [75] and later with fully automated analysis by Ebel et al. [76]. A comparison between EPSI and conventional MRSI spectra indicated a similarity in SNR per unit volume and unit time [60, 77]. However, an outstanding feature of the two-dimensional EPSI method [55] is the improvement of spatial resolution and SNR for a number of metabolites at short TE (13 ms) and acquisition time (64 s). In addition to evaluating and detecting the three major metabolite maps (NAA, Cho, Cr), 2D-EPSI was also applied to measure the changes in brain lactate at long TE (272 ms) and 1.5Tesla [78].

**Fig. 9:**
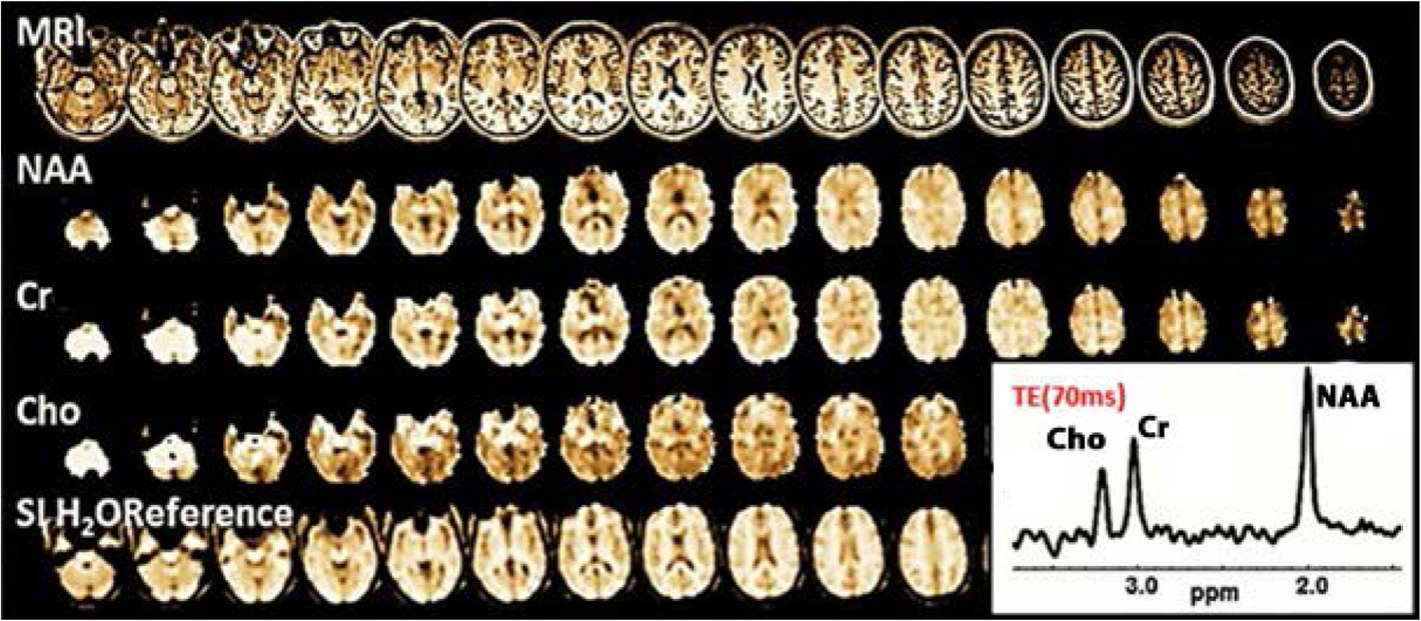
Whole brain mapping and a spectrum of major metabolites, mean water-reference spectroscopic imaging (SI H_2_O Reference) using EPSI at 3 T from a healthy subject (TE/TR = 70/1710 ms), total acquisition time (26 min), k-space points (50 × 50 × 18), FOV (28 × 28 × 18 cm^3^) and voxel volume (0.31 cm^3^). Reproduced with permission from [48]

3D-EPSI was implemented by Maudsley et al. [79] in mapping the distributions of the three major metabolites (NAA, Cho, Cr) over a wide region of the human brain at intermediate TE (70 ms) where metabolite ratios and average metabolite values in GM and white matter (WM) were clinically determined on a 3 T MRI scanner. MRSI data processing was carried out by a fully automated processing approach (Metabolite Imaging and Data Analysis System (MIDAS)) [80]. Metabolite maps obtained from volumetric EPSI technique with an acquisition time of 26 min are shown in Fig. 9.

New EPSI methods were developed where the quantity of k-space lines are reduced. When 2D-spatial selective RF (2DRF) are incorporated within EPSI sequences, a new type of 2DRF-EPSI is obtained [81]. 2DRF-EPSI addresses the poor image quality that results from artefacts and low spatial resolution, by shortening echo-train length, and doubling the spatial resolution along the direction of phase-encoding.

The implementation of EPSI techniques at high field (3 to 7 T) has enabled not only to linearly gain SNR per unit volume and time but has also allowed for the evaluation of J-coupled metabolites such as glutamate (Glu) and glutamine (Gln) [48]. 3D-EPSI was successfully applied to assess the concentrations of major metabolites, including J-coupled, at 4 T and 3 T in GM and WM [53] of healthy volunteers. This is an important development as it has greatly increased the spectral resolution and SNR associated with shortened experimental time (<10 min) and has thus sparked interest in clinical studies of MS and stroke for the potential benefits of this methodology [53].

Short TE EPSI was recently introduced by Ding et al. [82] to evaluate the neurochemical variation of major metabolites as well as Glx and mI in conjunction with parallel imaging acquisition. NAA, tCr, Cho, Glx and mI were found to have different mapping concentrations in WM and GM in comparison with other short TE (15 ms) studies [49]. Mapping of whole brain metabolites was also achieved by implementation of 3D-EPSI at short TE (20 ms) [83]. An improvement in short TE EPSI applications with high spatial resolution and improved SNR was increasing spatial sensitivity using multiple coils [84, 85].

The significant development of advanced gradient hardware has resulted in the emergence of a new EPSI method that focuses on high spatial resolutions with a large coverage of the human brain at 3 T. The flyback 3D-EPSI technique [40] was presented to improve the spatial resolution and SNR for different metabolites (NAA, Cr, Cho and lactate). Zierhut et al. employed flyback EPSI for a detailed analysis of the data from a human glioma patient with an acquisition time of less than 9.5 min, with a spatial resolution of 1 cc [40].

The developments in whole brain coverage have shown that the efficiency of spatial and spectral encoding can be improved by applying volumetric EPSI techniques. However, these improvements are still limited by long acquisition times, which are considered to be a crucial factor in many clinical studies [86]. The first modification to enhance the acceleration of data collection was the use of SENSE-EPSI. This strategy combined the spatial and spectral encoding capabilities and has been investigated by Lin et al. [87] to obtain major brain metabolites maps. In this particular study, the data acquisition time was halved to 32 s for 32 × 32 image matrix with high spatial-temporal resolution, using SENSE acceleration factor of two. However, SNR declines with faster acceleration, which can affect the usefulness of these techniques clinically. 3D-EPSI and 2D-SENSE [49] are combined to acquire higher spatial resolution data that covers the whole brain in a shorter acquisition time (1 min) for 32 × 32 × 8 spatial matrix and TE (15 ms) at high field (3 T).

Another method that can be used to map metabolite distribution in the whole brain is the 3D GRAPPA-EPSI techniques [88]. The spectral quality, brain metabolite concentrations and SNR values from 3D GRAPPA-EPSI were obtained with an acceleration factor of 1.5 shows similar results to the 3D-EPSI technique [88]. Reduction of SNR has become a major challenge for implementing 2D GRAPPA-EPSI [89] techniques with a 32 channel coil array [53], which improves SNR values due to the large numbers of small sized coils [90]. In addition to this, 2D GRAPPA-EPSI allows for the mapping of most metabolites within a much shorter time.

Dydak et al. incorporated the MEscher-GArwood (MEGA) editing scheme [91] within the EPSI technique [92, 93] for mapping of the main inhibitory neurotransmitter γ-aminobutyric acid (GABA) levels. The MEGA-EPSI method can perform data acquisition of GABA level activity in less than 10 min in a 2D slice. The short acquisition time and high sensitivity of the 2D MEGA-EPSI lead to the creation of 3D MEGA-EPSI technique due to its increased spatial resolution with an acquisition times of 17 min for eight slices at 3 T, which is a major improvement compared to other techniques [94].

**Fig. 10:**
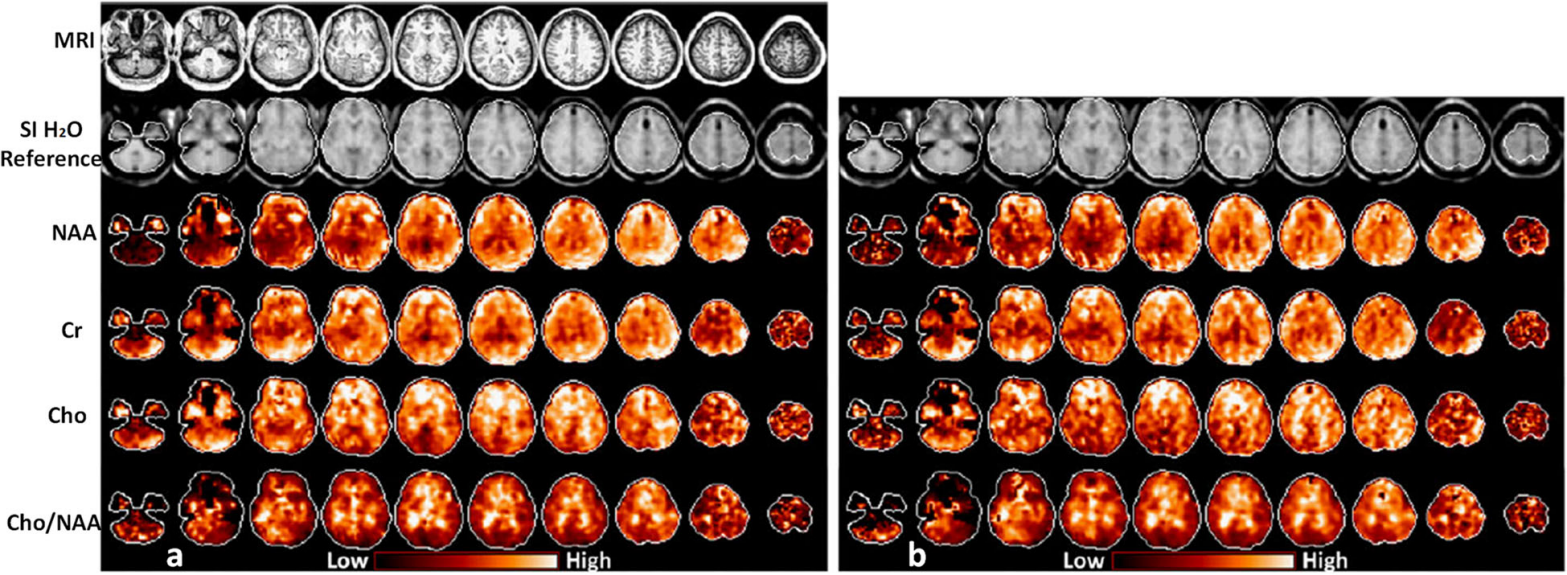
Illustrates the comparison between two fast MRSI sequences at 3 T: **a** the 3D-EPSI and **b** GRAPPA-EPSI sequences, to show the whole brain mapping of metabolites with interleaved water reference acquisition (SI H2O Reference) from a healthy normal subject at intermediate TE (70 ms). Reproduced with permission from [84]

Recently, image quality and brain metabolites concentrations have been studied by applying a commonly reduced k-space strategy at 3 T. For this purpose, the GRAPPA-EPSI technique was introduced by Sabati et al. [84] to improve the spectral quality associated with accelerated acquisition of volumetric EPSI data. This has resulted in an of experimental time of 16 min at the expense of SNR values [40]. The results obtained from 3D-EPSI techniques are compared to the GRAPPA-EPSI technique in Fig. 10.

A further benefit to the EPSI technique is its flexibility to adapt to a wide range of techniques to improve speed of data collection in certain specialised areas across a wide-range of MRSI and MRI techniques, including: Flyback, GRAPPA, and SENSE in 2D and 3D modes, that would otherwise be relying on slow conventional MRSI methods.

Some disadvantages regarding EPSI need to be mentioned. The speed of data collection is the root cause for EPSI’s major technical problems with the gradient system, especially when recording data with disequilibrium of positive and negative gradient lobes [38]. This leads to further contraction of spectral bandwidth which poses a problem considering EPSI has less SNR than traditional phase-encoded MRSI. Therefore, multiple averages are required to improve SNR. Regardless, when the above challenges are suitably addressed, EPSI can be considered one of the best techniques for whole brain 3D-MRSI [76].

#### Comparison of MRSI techniques

Advances on MRSI techniques have focussed on either improving the temporal resolution or investigating the relationship between spatial resolution and SNR. To achieve these aims, work has been carried out to improve the MRSI techniques and increase spatial coverage e.g. 2D-3D MRSI.

Detecting various brain metabolites in vivo, using different parameters for 3D PRESS-MRSI [36, 40], showed variable NAA concentrations in different acquisitions at 3 T. Recently, the 3D PRESS-MRSI has been improved by using 4 slices in PRESS box and outer volume suppression pulses to cover the whole brain with an acquisition time of 9 min, leading to spectral data of NAA, Cho and Cr [37]. Multiple 2D-MRSI has significant improvements for spatial resolution, SNR and whole brain metabolite mapping at long TE [95].

A summary of the results of various MRSI methods are shown in Table 1. Data shown represents measured metabolites from healthy controls (HCs) using PRESS-MRSI with different parameters.

**Table 1:** Brain metabolite concentration and acquisition time of various MRSI techniques

Methods	TE/TR (ms)	Metabolite (mM)	Tacq (min)^a^	Brain region/ FOV/ voxel size/B_o_ (Tesla)	Ref.
3D PRESS-MRSI	144/1100	NAA: 10.1 ± 0.64	21.12	Centre of brain/ 12 × 12 × 8 cm^3^/1 cm^3^/3 T	[40]
3D PRESS-MRSI	144/1100	NAA: 6.9 ± 1.3, Cho: 6.5 ± 1.4 Cr: 6.1 ± 1.2	17	Not reported/ 12x12x8 and 16x16x8 cm^3^/1 cm^3^/3 T	[36]
3D PRESS-MRSI	144/1100	Cho/NAA:(0.51–0.54)	21.12	Not reported/ 12 × 12 × 8 cm^3^/ 1 cm^3^/3 T	[46]
Multiple 2D-MRSI	144/ 2300	NAA:2.5 ± 4.47, Cho:2.94 ± 9.71, Cr:7.3 ± 4.09	26	Whole brain scans/ Not reported	[95]
2D-MRSI	135/1500	MS patients: NAA:9.48 ± 0.73 Controls: NAA:11.58 ± 0.37	NR^b^	WM and GM/ 16 × 16 cm^2^/ 2 cm^3^/1.5 T	[112]
3D PRESS-MRSI with OVS	144/1500	Cho/Cr :1.24 NAA/Cr:1.94	9	Whole brain/ 16 × 16 × 16, 18x18x16 cm^3^/1 cm^3^/3 T	[37]

*Abbreviations*: ^a^ *Tacq* acquisition time, ^b^ *NR* Not reported

Improvements in spatial coverage and temporal resolution have been achieved by using novel MRSI techniques as shown in Table 2. High speed EPSI was used at short TE (15 ms) to find out that in HCs Glu (12.8 ± 1.5 mM) in GM is of a significantly higher concentration than in WM (7.0 ± 1.1 mM) and also higher than other brain metabolites like NAA (8.6 ± 0.7 mM) and mI (6.3 ± 0.7 mM) in GM in HCs [53]. These improvements in temporal resolution due to GRAPPA enabled by higher number of coil elements (32 channel) [89] make this technique suitable for clinical studies with acceptable acquisition times. Whole brain has also been studied by using EPSI techniques at short TE (17.6 ms) to measure the brain metabolite in both GM and WM of brain. The results showed that the value of NAA concentration (12.05 ± 0.47 mM) is higher in the parietal lobes in GM than NAA concentration (8.74 ± 0.34 mM) in WM [82].

**Table 2:** Brain metabolite concentrations obtained in fast MRSI techniques

Methods	TE/TR (ms)	Metabolite (mM)	Tacq (min)	Brain region/ FOV/ voxel size/ Cohort/ Bo (Tesla)	Ref
EPSI	18/1550	GM in frontal lobe left: NAA:11.10 ± 0.32, tCho:2.04 ± 0.16, Glx:10.22 ± 0.53 WM in frontal lobe left: NAA:9.51 ± 0.27, tCho:1.95 ± 0.16, Glx:6.43 ± 0.34	16	whole brain/ 28 × 28 × 18 cm^3^/1 cm^3^/ 20 HCs/ 3 T	[82]
EPSI with OVS	15/2000	GM: NAA:8.6 ± 0.7, Glu:12.8 ± 1.5, tCho:1.4 ± 0.2. WM: NAA:7.2 ± 0.7, Glu:7.0 ± 1.1, tCho:1.4 ± 0.2	8.5	WM and GM/ 26 cm/ 1 cm^3^/ 9 HCs/ 3 T	[53]
3D GRAPPA-EPSI	70/1710	^a^NAA: 595 ± 37.9, Cr: 346 ± 23.9 Cho: 100 ± 9.7	16	Whole brain/ 28 × 28 × 18 cm^3^/ 0.31 cm^3^/ 25 patients with mild traumatic brain injuries (mTBI) & 25 HCs/ 3 T	[84]
GRAPPA-EPSI	15/2000	GM: NAA:15.36 ± 2.62, Glx:18.40 ± 3.19, Cho:3.19 ± 0.58 WM: NAA: 14.46 ± 1.79, Glx: 11.01 ± 3.20, Cho:3.19 ± 0.45	1.5	WM and GM/ 24 cm/ 0.85 cm^3^/ 5 HCs/ 3 T	[89]
3D SENSE-EPSI	15/2000	NAA:9.5 ± 4.3, Cho:1.4 ± 0.9, Glu:8.8 ± 5.2, Cr:7.2 ± 2.8	8.5	WM and GM/ 24 × 24 × 10 cm/ 0.7 cm^3^/ HCs/ 3 T	[49]
3D EPSI-MRSI	70/1710	^b^GM: NAA:4948 ± 75, Cr: 3461 ± 53, Cho: 544 ± 13 in Occipital WM: NAA:5165 ± 81, Cr: 3048 ± 48, Cho: 671 ± 12 in Occipital	NA	WM and GM/ 28 × 28 × 18 cm^3^/ 0.31 cm^3^/ 88 HCs/ 3 T	[79]
3D PRESS-MRSI	144/1500	Concentration for age groups (25–32) NAA/Cr:1.8 in Frontal lobe NAA/Cr : 1.9 in Occipital lobe	13	Whole brain/ 32 cm /1 cm^3^/ 8 HCs/ 1.5 T	[61]

^a^Mean metabolite concentrations averaged over all voxels of WM brain tissue in parietal lobe

^b^Average metabolite values in IU

Whole brain was also studied by GRAPPA-EPSI short TE technique, where NAA (15.36 ± 2.62), mI (6.11 ± 1.14), tCr (11.97 ± 1.67) and Glx (18.40 ± 3.19) mM where found to be more abundant in GM than WM [89]. In addition, GRAPPA-EPSI sequence at TE of 70 ms [84] found that WM NAA concentration to be (595 ± 37.9) which is higher than Cr (346 ± 23.9) and Cho (100 ± 9.7) institutional units (IU) for HCs. A summary of fast MRSI studies in human brain and their results are shown in Table 2.

### MRSI in multiple sclerosis

MRSI was applied to MS patients at 1.5 T with long TE values [96–99] and at 2 T with short TE values [100]. Some studies focused on lesions compared to NAWM and GM [14]. Other studies compared distinct different clinical groups such as RRMS [101, 102], SPMS and PPMS [103, 104].

Conventional MRSI techniques have identified changes in metabolite concentration in limited regions within NAWM or GM affected by the disease process [105–107]. For this purpose, Tiberio et al. used 3D PRESS-MRSI at TE of 30 ms and 1.5 T. Statistically significant metabolic differences between RRMS patients and HCs were found in NAWM total NAA and cortical gray matter (CGM) Glx and Cho [107]. This technique was also used at TE of 40 ms and 3 T by Ratiney et al. [108] to investigate mI concentration level in normal appearing white and gray matter (NAGM). mI concentration was significantly increased in both NAWM and NAGM of MS patients compared to control subjects. Changes in the above metabolites have been associated with clinical impairment or/and neuronal dysfunction in MS patients.

In another study, RRMS patients and age-matched controls were recruited to investigate abnormal metabolic changes in GM and WM by using conventional 3D-MRSI techniques at 3 T [102]. With an MRSI scan lasting 34 min, it was found that the concentrations of Cr, Cho and mI in WM of RRMS group were higher (p ≤ 0.01) compared to controls, while WM NAA was lower (*p* = 0.07). NAA reduction reflects neuronal and glial loss or impairment at early stage of RRMS, whereas brain inflammation leads to increase of mI and Cr concentration levels due to intense gliosis. Additionally, increase in Cho levels is usually associated with abnormal membrane turnover from myelin breakdown.

Suhy et al. [104] found that NAA was reduced in RRMS and PPMS from NAWM compared to HCs WM. Similarly, the normalized value of brain metabolites in NAWM of RRMS and PPMS patients shows that there is a significant decrease in NAA/Cr ratio (*p* = 0.027). These results were achieved by applying a 2D PRESS-MRSI at long TE (135 ms) at 1.5Tin RRMS and PPMS groups. However, by applying a 2D PRESS-MRSI at TE (135 ms) at 3 T no statistically significant difference of absolute concentrations of Cr, NAA and NAA/Cr ratio was found in NAWM between RRMS vs control group [109], but significant reduction was found for NAA/Cr (*p* < 0.0003 and *p* < 0.001) in SPMS compared to RRMS and control groups. The above work suggested reduction in NAA and NAA/Cr as potential indicators for irreversible damage in MS. NAA Reduction is usually associated with neuroaxonal injury. Astrocytic proliferation reflects increased levels of Cr. NAA and NAA/Cr are constantly reported with lower level values from NAWM and lesion of MS patients. Recently, Khan et al. showed that tNAA/tCr (1.88 ± 0.19 mM, *p* < 0.05) has strong and statistically significant inverse correlation with total disability score in a group of 39 RRMS patients [110].

Spectroscopic data from early RRMS in NAWM, CGM and lesions have been reported by Kapeller et al. [111]. This study focused primarily on quantifying the concentration of the NAA, Cr and mI using PRESS-MRSI technique at short TE (30 ms). Lower NAA concentration (*p* < 0.01) was found in RRMS in CGM and NAWM compared to controls. Shorter TE is more sensitive to coupled metabolites than longer TE spectroscopy.

**Fig. 11:**
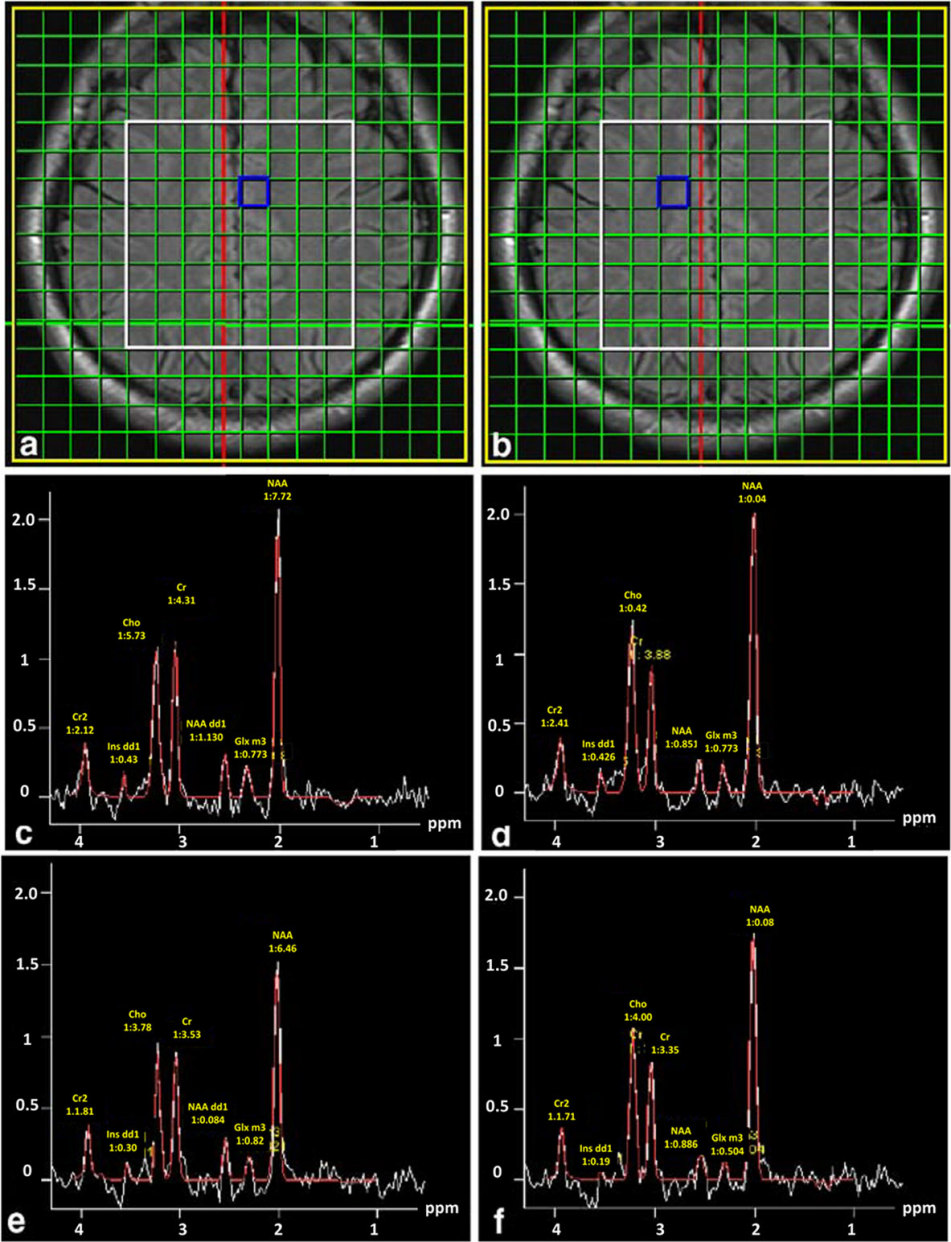
Shows the spectra of voxels in **a** GM and **b** WM of healthy and PPMS subjects: **c**, **d** acquired from healthy subject in GM and WM, respectively and **e**, **f**: acquired from GM and WM regions of a PPMS patient, respectively. Experimental parameters: TE/TR = 135 ms/1500 ms, FOV (16 × 16 cm^2^, *yellow border*) and VOI (8 × 8 × 2 cm^3^, *white border*). Reproduced with permission from [112]

A PPMS group was studied by Sijens et al. at long TE (135 ms) by using 2D PRESS-MRSI [112]. Results from the brain metabolic maps from PPMS patients confirmed that Cr levels were elevated, whereas NAA and Cho concentrations were decreased in WM more than in GM in PPMS compared with controls. The bigger decrease of NAA and Cho in WM is expected as a result of the larger presence of myelin in WM than in GM [112]. Figure 11 depicts brain sample spectral data from PPMS patients and controls in GM and WM [112].

Recently, in vivo proton MRSI technique at long TE (135 ms) and 3 T has been reported by Rahimian et al. [113] not only to measure brain metabolite concentration of NAA, Cho and Cr, NAA/Cr and NAA/Cho in RRMS and PPMS groups but also to differentiate between MS subtypes. Comparing the results obtained shows that there was a significant increase in Cr concentration in non-enhancing lesions of RRMS patients compared to the PPMS patients (*p* = 0.008) whilst the concentration of NAA/Cr was significantly reduced (*p* = 0.03) in PPMS compared with RRMS. However, there was no statistically significant difference in concentrations of Cho, NAA and NAA/Cho between the two groups.

**Fig. 12:**
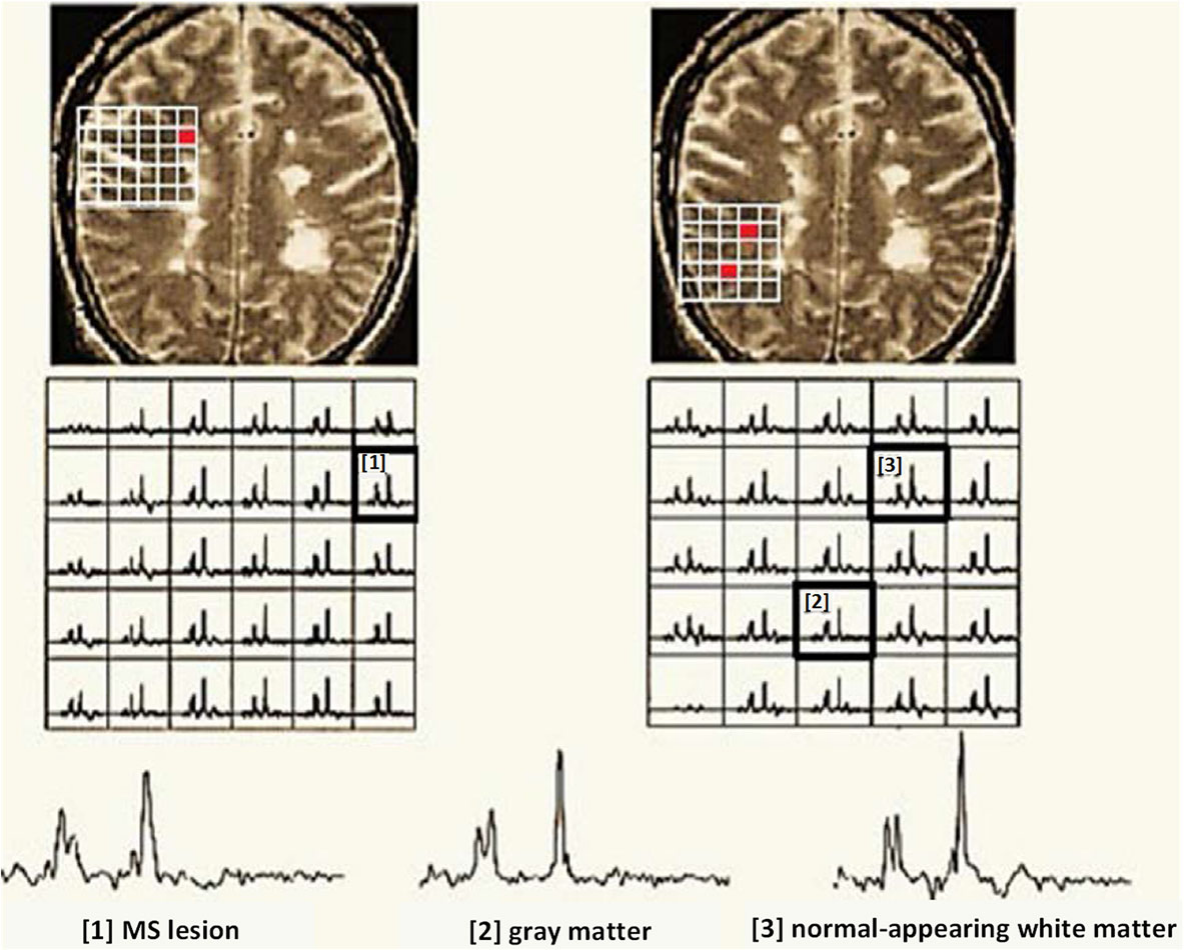
Spectroscopic data from a typical SPMS brain from three separate regions: [1] *MS lesion*, [2] *CGM* and [3] *NAWM*. The voxels are represented by the numbers and *red spots*. Experimental parameters: 3D-EPSI (TE/TR = 144/2000 ms), acquisition time (20 min), FOV (24 × 24 × 8 cm^3^) and slice thickness (4 mm) at 1.5 T. Reproduced with permission from [114]

EPSI allowed detection of neuro-metabolites in the visual tract WM of MS patients [5]. In this study, EPSI was used to study neurochemical changes at 1.5 T associated with visually evoked potential (VEP) abnormalities. It was found that the average value of NAA is significantly lower (*p* ≤ 0.05) in the abnormal VEP group than in the normal VEP group. 3D-MRSI was implemented using EPSI techniques at 1.5 T [114] to measure the major metabolite ratios in control and MS cohorts from two ROIs: supratentorial brain and central brain. This study was performed at long TE (144 ms) on three subtypes of MS: RRMS, PPMS and SPMS, where it was found that NAA/Cr ratio was significantly decreased (*p* < 0.01) for all MS cohorts compared to HCs in both ROI. Sample spectroscopic data from SPMS patients are shown in Fig. 12.

The multi-slice EPSI technique was introduced by Mathiesen et al. [86] as a response to the limitations of VOI in conventional MRS techniques. The introduction of this technique made it possible to measure brain metabolites in specific regions such as MS lesions, NAWM, CGM and to estimate WBNAA which is generally considered an indicator of disease progression and treatment efficiency. Decreases in global ratios (NAA/Cr and Cho/Cr) were noticed for early MS patients compared with healthy subjects, however, no statistically significant differences were noticed in NAA/Cr between these two groups [86].

Due to the capabilities of multi-slice EPSI technique to cover larger regions of the brain, it has been employed to evaluate the global brain NAA/Cr ratio, which might be a good indicator for progression and cognitive decline in MS [115]. Thus, measuring NAA/Cr ratio in WM and GM within scan times of 20 min and TE of 144 ms, provided multi-slice EPSI a particular clinical utility. Similarly, reduction of NAA in GM of RRMS and SPMS groups was demonstrated by using a volumetric spiral MRSI technique [4] at a low static field of 1.5 T. A study by De Stefano et al. confirmed that brain MR spectroscopic data has a significant role in detecting neuoraxonal damage caused by MS disease with decreased NAA/Cr ratio [116] due to the consistent finding of decrease NAA signal being linked to axonal damage. Moreover, acute lesions were found to have high Glu levels; which might suggest a connection between the axonal injury in active lesions and Glu excitotoxicity [116]. Donadieu et al. used fast 3D-EPSI (TE: 20 ms) at 3 T to acquire spectral maps of the complete brain of MS patients (*N* = 19) and compared them to age and gender matched HCs [117]. Their findings indicated a reduction in NAA and Glx (−15 and −20%) in GM, while mI was increased in WM of MS group (+22%). A summary of the results of MRSI methods are shown in Table 3.

**Table 3:** Brain metabolite concentration were acquired for MS patients and HCs by applying PRESS-MRSI and fast MRSI methods

Methods	TE/TR (ms)	Metabolite (mM)	Tacq ^a^ (min)	Brain region/ FOV/voxel size/Cohort/Bo (Tesla)	Ref
2D-MRSI	30/3000	CGM: Controls, tNAA: 8.3 ± 1.0, Cr: 5.9 ± 0.8 RRMS, tNAA: ↓, Cr: ↓	29	NAWM, CGM/ 30 cm/2.34 mL/ 16 RRMS, 12 HCs/1.5 T	[111]
2D-MRSI	135/1800	Controls, NAA/Cr: 2.22 ± 0.19 RRMS, NAA/Cr: ↓ PPMS, NAA/Cr: ↓	13	WM, NAWM/ 21 × 21 cm/ 2.4 cm^3^/ 15 PPMS, 13 RRMS & 20 HCs/ 1.5 T.	[104]
PRESS-MRSI	30/3000	NAWM: Controls, tNAA: 8.82, Cr: 4.87 RRMS, tNAA: ↓, Cr : ↓		NAWM, CGM/ 30x30 cm/ 2.3 mL/ 25 RRMS, 28 HCs/ 1.5 T	[105]
2D-MRSI	135/1500	GM: Controls, NAA:10.63 ± 0.6, Cr:6.13 ± 0.31 PPMS, NAA: ↓, Cr: ↓ WM: Controls, NAA:11.48 ± 0.37, Cr: 5.35 ± 0.45 PPMS, NAA: ↓, Cr: ↓	7	WM and GM/ 16 ×16 cm^2^/ 2 cm^3^/ 4 PPMS & 4 HCs/ 1.5 T	[112]
PRESS-MRSI	30/3000	NAWM: Controls, tNAA: 9.18 ± 0.6 RRMS, tNAA: ↓ CGM: Controls, tNAA: 9.28 ± 1.02 RRMS, tNAA: ↓	NA	NWWM and CGM/ 30x30 cm/ 20 RRMS, 10 HCs /1.5 T	[107]
3D-MRSI	70/1710	GM: Controls, NAA:8.5 ± 0.6, Cr: 6.8 ± 0.6 RRMS, NAA: ↑, Cr: ↑ WM: Controls, NAA:7.9 ± 0.6, Cr: 4.9 ± 0.2 RRMS, NAA: ↓, Cr: ↑	34	WM and GM/ 16x16 cm/ 0.75 cm^3^, 18 RRMS, 10 HCs/ 3 T	[102]
MRSI	135/1000	RRMS, NAA: 8.45 ± 0.88, Cr: 5.2 ± 0.73 PPMS, NAA: ↓, Cr:↑	45	MS lesion for RRMS & PPMS/ 16×16/ 1.2 cm^3^/ 15 RRMS, 15 PPMS/ 3 T	[113]
2D PRESS-MRSI	135/1500	NAWM: Controls, NAA: 12.3 ± 0.4, Cr: 8.4 ± 0.4 RRMS, NAA: ↑, Cr: ↑ SPMS, NAA: ↓, Cr: ↓	15	Fronto-parietal WM/ 16x16 cm/1 cm^3^/ 27 RRMS, 10 SPMS & 8 HCs/ 3 T	[109]
EPSI	272/4000	Normal VEP, NAA: 7692 ± 632 Abnormal VEP NAA: ↓	9	Mid-thalamus / 24 cm/ 9 MS with abnormal VEP& 8 MS normal VEP/ 1.5 T	[5]
3D-EPSI	144/2000	Central brain: Controls, NAA/Cr: 1.963 ± 0.167 MS patients, NAA/Cr: ↓	20	Corpus callosum, STB/ 24x24x16 cm/ 1 cm^3^/ 9 RRMS, 21 SPMS, 18 PPMS & 10 HCs/ 1.5 T	[114]
EPSI	144/4300	NAA/Cr: 1.55 ± 0.1 from RRMS	20	Whole brain/ not reported /1 cm^3^, 20 RRMS & 75 HCs/ 1.5 T	[115]
Spiral-MRSI	144/2000	WM: Controls, NAA: 159 A.U. (mean conc.) RRMS, NAA: ↓ SPMS, NAA: ↓	15	NAWM, GM in supratentorial brain/ 24 cm/ 1.2 mL/ 5 RRMS, 5 SPMS & 9 HCs/ 1.5 T	[4]
EPSI	144/4300	Controls, NAA/Cr: 1.5 ± 0.09 MS patients, NAA/Cr: ↑	20	Whole brain/ not reported/ 1 cm^3^, 18 RRMS & 18 HCs/ 1.5 T	[86]
3D-EPSI	20/1720	GM: Controls, NAA: 10 ± 0.85, Glx: 6.5 ± 1.1 MS patients, NAA: ↓, Glx: ↓	17.48	Whole brain/ 28×28×18 cm/ 1 cm^3^, 19 RRMS & 19 HCs / 3 T	[117]

^a^ *Tacq* acquisition time

### Reproducibility of MRSI of MS patients

Reproducibility of fast MRSI techniques has not been evaluated. It is important that MRSI techniques have good reproducibility and recent studies have shown good correlation between time points. Mostert et al. tested MRSI reproducibility with stable MS patients 4 week apart and found good short term reproducibility of NAA/Cr measurement [118]. Vafaeyan et al. also validated the reproducibility of NAA/Cr and NAA/Cho in MS as they found high correlation of these ratios at different time points [119].

## Conclusions

In recent years, the evolution of MR technologies has led to developments of brain ^1^H-MRS and clinical MR imaging procedures which enabled the collection of morphometric and biochemical information in a single imaging session. There are a variety of ^1^H-MRS techniques that are used to detect and evaluate the biochemical changes of whole brain, where whole brain coverage requires improving spatial resolution and SNR. Fast MRSI is more suited to a clinical setting, mainly due to faster acquisition time, improved SNR and spatial resolution.

The short acquisition time of fast MRSI requires advanced and robust design in the hardware and software systems to improve and modify sequences for these techniques, making them more adaptable, especially when there is a need to combine them with other acceleration techniques by either increasing field strength or modifying coil design.

EPSI has brought about revolutionary improvement in the technological capacity of MRSI, compared to conventional MRSI methods. This development has improved the time efficiency of data collection and enhanced the recording brain metabolite distribution. Data acquisition has seen the biggest improvement, with speeds more than two to three times faster than that of traditional MRSI methods. This has provided the opportunity for clinical research to be done on whole brain in acquisition times of less than 16 min.

This article has shown the benefits and ability of the EPSI to adapt to a diverse range of techniques, leading to: flyback-EPSI, MEGA-EPSI, GRAPPA-EPSI, SENSE-EPSI ^1^H-MRSI techniques and 2D and 3D echo-planar techniques. This also enables the detection of J-coupled brain metabolites like: GABA, Glu, Gln and mI. Moreover, it can be concluded that the echo-planar imaging sequence has proven to be a successful and accelerated technique in clinical studies not only in multiple sclerosis [4] but also in Parkinson’s disease [1], Alzheimer’s disease [2] and stroke [3].

In vivo spectroscopy has the potential to play an important role in biomarker discovery and disease activity prediction in MS. MRSI studies confirmed that NAA/Cr can distinguish MS from HCs independent from the ROI. NAA can also be a potential marker of neuronal function in NAWM, CGM for different clinical groups identifying the progressive stage of the disease. Longitudinal studies with techniques applicable in clinical setting are required to determine if MRSI can close the gap in MRI imaging by defining the disease course for the individual patient. The larger ROI afforded by MRSI compared to conventional MRS has provided evaluations of neurometabolite changes to be assessed across a much larger area, thereby assessing whole brain pathological changes occurring in MS. The improvements in achieving shorter acquisition times for MRSI now provide a greater likelihood of clinical application of these techniques.

## Abbreviations

1D: One dimensional
2D: Two dimensional
3D: Three dimensional
Bo: Static magnetic field
CGM: Cortical gray matter
CHESS: Chemical shift selective suppression pulses
Cho: Choline
Cr: Creatine
EPI: Echo planar imaging
EPSI: Echo planar spectroscopic imaging
FOV: Field of view
GABA: γ-Aminobutyric acid
Gln: Glutamine
Glu: Glutamate
Glx: Glutamine + glutamate
GM: Gray matter
GRAPPA: Generalised autocalibrating partially parallel acquisitions
HC: Healthy control
Lac: Lactate
MEGA: MEscher-GArwood
mI: Myo-inositol
mM: Millimolar
MRI: Magnetic resonance imaging
MRS: Magnetic resonance spectroscopic
MRSI: Magnetic resonance spectroscopic imaging
ms: Millisecond
MS: Multiple sclerosis
NAA: N-acetylaspartate
NAGM: Normal appearing gray matter
NAWM: Normal appearing white matter
OVS: Outer volume suppression
ppm: Parts per million
PPMS: Primary progressive multiple sclerosis
PRESS: Point resolved spectroscopy
PSF: Point spread function
RF: Radiofrequency
ROI: Region of interest
RRMS: Relapsing-remitting multiple sclerosis
s: Second
SENSE: Sensitivity encoding
SMASH: Simultaneous acquisition of spatial harmonics
SNR: Signal-to-noise ratio
SPMS: Secondary progressive multiple sclerosis
STEAM: Stimulated echo acquisition mode
SVS: Single-voxel spectroscopy
T: Tesla
Tacq: Acquisition time
tCho: Total choline
tCr: Total creatine
TE: Echo time
TM: Mixing time
TR: Repetition time
VOI: Volume of interest
WBNAA: Whole brain N-acetylaspartate
WM: White matter

## References

[1] Levin BE, Katzen HL, Maudsley A, Post J, Myerson C, Govind V, Nahab F (2014). Whole-brain proton MR spectroscopic imaging in Parkinson’s disease. J Neuroimaging.

[2] Mandal PK (2007). Magnetic resonance spectroscopy (MRS) and its application in Alzheimer’s disease. Concepts Magn Reson Part A.

[3] Graham GD, Blamire AM, Howseman AM, Rothman DL, Fayad PB, Brass LM, Petroff OA (1992). Proton magnetic resonance spectroscopy of cerebral lactate and other metabolites in stroke patients. Stroke.

[4] Adalsteinsson E, Langer-Gould A, Homer RJ, Rao A, Sullivan EV, Lima CA, Pfefferbaum A (2003). Gray matter N-acetyl aspartate deficits in secondary progressive but not relapsing-remitting multiple sclerosis. AJNR Am J Neuroradiol.

[5] Heide AC, Kraft GH, Slimp JC, Gardner JC, Posse S, Serafini S, Bowen JD (1998). Cerebral N-acetylaspartate is low in patients with multiple sclerosis and abnormal visual evoked potentials. AJNR Am J Neuroradiol.

[6] Bottomley PA, Edelstein WA, Foster TH, Adams WA (1985). In vivo solvent-suppressed localized hydrogen nuclear magnetic resonance spectroscopy: a window to metabolism?. Proc Natl Acad Sci U S A.

[7] Frahm J, Bruhn H, Gyngell ML, Merboldt KD, Hanicke W, Sauter R (1989). Localized high-resolution proton NMR spectroscopy using stimulated echoes: initial applications to human brain in vivo. Magn Reson Med.

[8] Bertholdo D, Watcharakorn A, Castillo M (2013). Brain proton magnetic resonance spectroscopy: introduction and overview. Neuroimaging Clin N Am.

[9] Ordidge RJ, Gordon RE, Methods and apparatus of obtaining NMR spectra. 1985. United States Patents: US. Patent number: RE32748. Date of Patent: Sep 13, 1988.

[10] Bottomley PA (1987). Spatial localization in NMR spectroscopy in vivo. Ann N Y Acad Sci.

[11] Brown TR, Kincaid BM, Ugurbil K (1982). NMR chemical shift imaging in three dimensions. Proc Natl Acad Sci U S A.

[12] Pykett IL, Rosen BR (1983). Nuclear magnetic resonance: in vivo proton chemical shift imaging. Work in progress. Radiology.

[13] Rovira A, Auger C, Alonso J (2013). Magnetic resonance monitoring of lesion evolution in multiple sclerosis. Ther Adv Neurol Disord.

[14] Sajja BR, Wolinsky JS, Narayana PA (2009). Proton magnetic resonance spectroscopy in multiple sclerosis. Neuroimaging Clin N Am.

[15] Kruger D (2012). Multiple sclerosis. JAAPA.

[16] Lublin FD, Reingold SC, Cohen JA, Cutter GR, Sorensen PS, Thompson AJ, Wolinsky JS (2014). Defining the clinical course of multiple sclerosis: the 2013 revisions. Neurology.

[17] Courtney SW. All About Multiple Sclerosis, M.D. Jack Burks, et al., Editors. New Jersey: Multiple Sclerosis Association of America; 2006.

[18] Polman CH, Reingold SC, Banwell B, Clanet M, Cohen JA, Filippi M, Fujihara K (2011). Diagnostic criteria for multiple sclerosis: 2010 revisions to the McDonald criteria. Ann Neurol.

[19] Traboulsee A, Simon JH, Stone L, Fisher E, Jones DE, Malhotra A, Newsome SD (2016). Revised Recommendations of the Consortium of MS Centers Task Force for a Standardized MRI Protocol and Clinical Guidelines for the Diagnosis and Follow-Up of Multiple Sclerosis. AJNR Am J Neuroradiol.

[20] De Stefano N, Bartolozzi ML, Guidi L, Stromillo ML, Federico A (2005). Magnetic resonance spectroscopy as a measure of brain damage in multiple sclerosis. J Neurol Sci.

[21] Muhlert N, Atzori M, De Vita E, Thomas DL, Samson RS, Wheeler-Kingshott CAM, Geurts JJG (2014). Memory in multiple sclerosis is linked to glutamate concentration in grey matter regions. J Neurol Neurosurg Psychiatry.

[22] De Stefano N, Filippi M, Miller D, Pouwels PJ, Rovira A, Gass A, Enzinger C (2007). Guidelines for using proton MR spectroscopy in multicenter clinical MS studies. Neurology.

[23] Caramanos Z, Narayanan S, Arnold DL (2005). 1H-MRS quantification of tNA and tCr in patients with multiple sclerosis: a meta-analytic review. Brain.

[24] Duarte JM, Lei H, Mlynarik V, Gruetter R (2012). The neurochemical profile quantified by in vivo 1H NMR spectroscopy. Neuroimage.

[25] Barkhof F (2002). The clinico-radiological paradox in multiple sclerosis revisited. Curr Opin Neurol.

[26] Ontaneda D, Fox RJ. Imaging as an outcome measure in multiple sclerosis. Neurotherapeutics. 2017;14(1):24–34. 10.1007/s13311-016-0479-6 PMC5233623 27699722

[27] Mader I, Roser W, Kappos L, Hagberg G, Seelig J, Radue EW, Steinbrich W (2000). Serial proton MR spectroscopy of contrast-enhancing multiple sclerosis plaques: absolute metabolic values over 2 years during a clinical pharmacological study. AJNR Am J Neuroradiol.

[28] Brief EE, Vavasour IM, Laule C, Li DK, Mackay AL (2010). Proton MRS of large multiple sclerosis lesions reveals subtle changes in metabolite T(1) and area. NMR Biomed.

[29] Gonen O, Catalaa I, Babb JS, Ge Y, Mannon LJ, Kolson DL, Grossman RI (2000). Total brain N-acetylaspartate: a new measure of disease load in MS. Neurology.

[30] Srinivasan R, Sailasuta N, Hurd R, Nelson S, Pelletier D (2005). Evidence of elevated glutamate in multiple sclerosis using magnetic resonance spectroscopy at 3 T. Brain.

[31] Gruber S, Pinker K, Riederer F, Chmelik M, Stadlbauer A, Bittsansky M, Mlynarik V (2008). Metabolic changes in the normal ageing brain: consistent findings from short and long echo time proton spectroscopy. Eur J Radiol.

[32] Drost DJ, Riddle WR, Clarke GD, Group AMT (2002). Proton magnetic resonance spectroscopy in the brain: report of AAPM MR Task Group #9. Med Phys.

[33] Garwood M, Delabarre L (2001). The return of the frequency sweep: designing adiabatic pulses for contemporary NMR. J Magn Reson.

[34] Bingolbali A, Fallone BG, Yahya A. Comparison of optimized long echo time STEAM and PRESS proton MR spectroscopy of lipid olefinic protons at 3 Tesla. J Magn Reson Imaging. 2015;41(2):481–486. 10.1002/jmri.24532 24338999

[35] Duijn JH, Matson GB, Maudsley AA, Weiner MW (1992). 3D phase encoding 1H spectroscopic imaging of human brain. Magn Reson Imaging.

[36] Li Y, Osorio JA, Ozturk-Isik E, Chen AP, Xu D, Crane JC, Cha S (2006). Considerations in applying 3D PRESS H-1 brain MRSI with an eight-channel phased-array coil at 3 T. Magn Reson Imaging.

[37] Ozhinsky E, Vigneron DB, Nelson SJ (2011). Improved spatial coverage for brain 3D PRESS MRSI by automatic placement of outer-volume suppression saturation bands. J Magn Reson Imaging.

[38] Barker PB, Lin DDM (2006). In vivo proton MR spectroscopy of the human brain. Prog Nucl Magn Reson Spectrosc.

[39] Zhu H, Barker PB (2011). MR spectroscopy and spectroscopic imaging of the brain. Methods Mol Biol.

[40] Zierhut ML, Ozturk-Isik E, Chen AP, Park I, Vigneron DB, Nelson SJ (2009). (1) H spectroscopic imaging of human brain at 3 Tesla: comparison of fast three-dimensional magnetic resonance spectroscopic imaging techniques. J Magn Reson Imaging.

[41] Spielman DM, Adalsteinsson E, Lim KO (1998). Quantitative assessment of improved homogeneity using higher-order shims for spectroscopic imaging of the brain. Magn Reson Med.

[42] Pruessmann KP, Weiger M, Scheidegger MB, Boesiger P (1999). SENSE: sensitivity encoding for fast MRI. Magn Reson Med.

[43] Sodickson DK, Manning WJ (1997). Simultaneous acquisition of spatial harmonics (SMASH): fast imaging with radiofrequency coil arrays. Magn Reson Med.

[44] Griswold MA, Jakob PM, Heidemann RM, Nittka M, Jellus V, Wang J, Kiefer B (2002). Generalized autocalibrating partially parallel acquisitions (GRAPPA). Magn Reson Med.

[45] Dydak U, Weiger M, Pruessmann KP, Meier D, Boesiger P (2001). Sensitivity-encoded spectroscopic imaging. Magn Reson Med.

[46] Banerjee S, Ozturk-Isik E, Nelson SJ, Majumdar S (2006). Fast magnetic resonance spectroscopic imaging at 3 Tesla using autocalibrating parallel technique. Conf Proc IEEE Eng Med Biol Soc.

[47] Bonekamp D, Smith MA, Zhu H, Barker PB (2010). Quantitative SENSE-MRSI of the human brain. Magn Reson Imaging.

[48] Posse S, Otazo R, Dager SR, Alger J (2013). MR spectroscopic imaging: principles and recent advances. J Magn Reson Imaging.

[49] Otazo R, Tsai SY, Lin FH, Posse S (2007). Accelerated short-TE 3D proton echo-planar spectroscopic imaging using 2D-SENSE with a 32-channel array coil. Magn Reson Med.

[50] Dydak U, Pruessmann KP, Weiger M, Tsao J, Meier D, Boesiger P (2003). Parallel spectroscopic imaging with spin-echo trains. Magn Reson Med.

[51] Srinivasan R, Cunningham C, Chen A, Vigneron D, Hurd R, Nelson S, Pelletier D (2006). TE-averaged two-dimensional proton spectroscopic imaging of glutamate at 3 T. Neuroimage.

[52] Kim DH, Henry R, Spielman DM (2007). Fast multivoxel two-dimensional spectroscopic imaging at 3 T. Magn Reson Imaging.

[53] Posse S, Otazo R, Caprihan A, Bustillo J, Chen H, Henry PG, Marjanska M (2007). Proton echo-planar spectroscopic imaging of J-coupled resonances in human brain at 3 and 4 Tesla. Magn Reson Med.

[54] Dager SR, Corrigan NM, Richards TL, Posse S (2008). Research applications of magnetic resonance spectroscopy to investigate psychiatric disorders. Top Magn Reson Imaging.

[55] Posse S, Tedeschi G, Risinger R, Ogg R, Le Bihan D (1995). High speed 1H spectroscopic imaging in human brain by echo planar spatial-spectral encoding. Magn Reson Med.

[56] Adalsteinsson E, Irarrazabal P, Topp S, Meyer C, Macovski A, Spielman DM (1998). Volumetric spectroscopic imaging with spiral-based k-space trajectories. Magn Reson Med.

[57] Paschal CB, Morris HD (2004). K-space in the clinic. J Magn Reson Imaging.

[58] Gagoski AB (2010). Spiral chemical shift imaging at 3 T using 32 channel receive array and online reconstruction. Prilozi.

[59] Block KT, Frahm J (2005). Spiral imaging: a critical appraisal. J Magn Reson Imaging.

[60] Pohmann R, von Kienlin M, Haase A (1997). Theoretical evaluation and comparison of fast chemical shift imaging methods. J Magn Reson.

[61] Gu M, Kim DH, Mayer D, Sullivan EV, Pfefferbaum A, Spielman DM (2008). Reproducibility study of whole-brain 1H spectroscopic imaging with automated quantification. Magn Reson Med.

[62] Delattre BM, Heidemann RM, Crowe LA, Vallee JP, Hyacinthe JN (2010). Spiral demystified. Magn Reson Imaging.

[63] Mayer D, Kim DH, Spielman DM, Bammer R (2008). Fast parallel spiral chemical shift imaging at 3 T using iterative SENSE reconstruction. Magn Reson Med.

[64] Bogner W, Gagoski B, Hess AT, Bhat H, Tisdall MD, van der Kouwe AJ, Strasser B (2014). 3D GABA imaging with real-time motion correction, shim update and reacquisition of adiabatic spiral MRSI. Neuroimage.

[65] Bogner W, Hess AT, Gagoski B, Tisdall MD, van der Kouwe AJ, Trattnig S, Rosen B (2013). Real-time motion- and B-correction for LASER-localized spiral-accelerated 3D-MRSI of the brain at 3 T. Neuroimage.

[66] Andronesi OC, Gagoski BA, Sorensen AG (2012). Neurologic 3D MR spectroscopic imaging with low-power adiabatic pulses and fast spiral acquisition. Radiology.

[67] Ozturk-Isik E, Chen AP, Crane JC, Bian W, Xu D, Han ET, Chang SM (2009). 3D sensitivity encoded ellipsoidal MR spectroscopic imaging of gliomas at 3 T. Magn Reson Imaging.

[68] Duyn JH, Moonen CTW (1993). Fast proton spectroscopic imaging of human brain using multiple spin-echoes. Magn Reson Med.

[69] Martin AJ, Liu H, Hall WA, Truwit CL (2001). Preliminary assessment of turbo spectroscopic imaging for targeting in brain biopsy. AJNR Am J Neuroradiol.

[70] Stengel A, Neumann-Haefelin T, Singer OC, Neumann-Haefelin C, Zanella FE, Lanfermann H, Pilatus U (2004). Multiple spin-echo spectroscopic imaging for rapid quantitative assessment of N-acetylaspartate and lactate in acute stroke. Magn Reson Med.

[71] Yahya A, Fallone BG (2009). Detection of glutamate and glutamine (Glx) by turbo spectroscopic imaging. J Magn Reson.

[72] Dydak U, Meier D, Lamerichs R, Boesiger P (2006). Trading spectral separation at 3 T for acquisition speed in multi spin-echo spectroscopic imaging. AJNR Am J Neuroradiol.

[73] Yang A, Xiao X, Wang Z (2014). Evaluation of normal changes in pons metabolites due to aging using turbo spectroscopic imaging. AJNR Am J Neuroradiol.

[74] Mansfield P (1984). Spatial mapping of the chemical shift in NMR. Magn Reson Med.

[75] Posse S, Decarli C, Le Bihan D (1994). Three-dimensional echo-planar MR spectroscopic imaging at short echo times in the human brain. Radiology.

[76] Ebel A, Soher BJ, Maudsley AA (2001). Assessment of 3D proton MR echo-planar spectroscopic imaging using automated spectral analysis. Magn Reson Med.

[77] Otazo R, Mueller B, Ugurbil K, Wald L, Posse S (2006). Signal-to-noise ratio and spectral linewidth improvements between 1.5 and 7 Tesla in proton echo-planar spectroscopic imaging. Magn Reson Med.

[78] Posse S, Dager SR, Richards TL, Yuan C, Ogg R, Artru AA, Muller-Gartner HW (1997). In vivo measurement of regional brain metabolic response to hyperventilation using magnetic resonance: proton echo planar spectroscopic imaging (PEPSI). Magn Reson Med.

[79] Maudsley AA, Domenig C, Govind V, Darkazanli A, Studholme C, Arheart K, Bloomer C (2009). Mapping of brain metabolite distributions by volumetric proton MR spectroscopic imaging (MRSI). Magn Reson Med.

[80] Maudsley AA, Darkazanli A, Alger JR, Hall LO, Schuff N, Studholme C, Yu Y (2006). Comprehensive processing, display and analysis for in vivo MR spectroscopic imaging. NMR Biomed.

[81] Rieseberg S, Frahm J, Finsterbusch J (2002). Two-dimensional spatially-selective RF excitation pulses in echo-planar imaging. Magn Reson Med.

[82] Ding XQ, Maudsley AA, Sabati M, Sheriff S, Dellani PR, Lanfermann H (2015). Reproducibility and reliability of short-TE whole-brain MR spectroscopic Imaging of human brain at 3 T. Magn Reson Med.

[83] Lecocq A, Le Fur Y, Maudsley AA, Le Troter A, Sheriff S, Sabati M, Donnadieu M (2015). Whole-brain quantitative mapping of metabolites using short echo three-dimensional proton MRSI. J Magn Reson Imaging.

[84] Sabati M, Zhan J, Govind V, Arheart KL, Maudsley AA (2014). Impact of reduced k-space acquisition on pathologic detectability for volumetric MR spectroscopic imaging. J Magn Reson Imaging.

[85] Kim DH, Gu M, Cunningham C, Chen A, Baumer F, Glenn OA, Vigneron DB (2009). Fast 3D (1)H MRSI of the corticospinal tract in pediatric brain. J Magn Reson Imaging.

[86] Mathiesen HK, Tscherning T, Sorensen PS, Larsson HB, Rostrup E, Paulson OB, Hanson LG (2005). Multi-slice echo-planar spectroscopic MR imaging provides both global and local metabolite measures in multiple sclerosis. Magn Reson Med.

[87] Lin FH, Tsai SY, Otazo R, Caprihan A, Wald LL, Belliveau JW, Posse S (2007). Sensitivity-encoded (SENSE) proton echo-planar spectroscopic imaging (PEPSI) in the human brain. Magn Reson Med.

[88] Zhu X, Ebel A, Ji JX, Schuff N (2007). Spectral phase-corrected GRAPPA reconstruction of three-dimensional echo-planar spectroscopic imaging (3D-EPSI). Magn Reson Med.

[89] Tsai SY, Otazo R, Posse S, Lin YR, Chung HW, Wald LL, Wiggins GC (2008). Accelerated proton echo planar spectroscopic imaging (PEPSI) using GRAPPA with a 32-channel phased-array coil. Magn Reson Med.

[90] Wiggins GC, Triantafyllou C, Potthast A, Reykowski A, Nittka M, Wald LL (2006). 32-channel 3 Tesla receive-only phased-array head coil with soccer-ball element geometry. Magn Reson Med.

[91] Mescher M, Merkle H, Kirsch J, Garwood M, Gruetter R (1998). Simultaneous in vivo spectral editing and water suppression. NMR Biomed.

[92] Dydak U, Marjanska M, Posse S. High-speed GABA mapping in human brain with MEGA-PEPSI at 3 Tesla. Proc Intl Soc Mag Reson Med. 2010;18:961.

[93] Dydak U, J. S. Xu, Marjanska M, Posse S. 3D GABA Spectroscopic imaging using MEGA-PEPSI. Proc Intl Soc Mag Reson Med. 2011;19:1428.

[94] Edden RA, Pomper MG, Barker PB (2007). In vivo differentiation of N-acetyl aspartyl glutamate from N-acetyl aspartate at 3 Tesla. Magn Reson Med.

[95] Dong Z, Liu F, Kangarlu A, Peterson BS (2012). Metabolite Mapping with Extended Brain Coverage Using a Fast Multisection MRSI Pulse Sequence and a Multichannel Coil. Int J Biomed Imaging.

[96] Arnold DL, Matthews PM, Francis GS, O’Connor J, Antel JP (1992). Proton magnetic resonance spectroscopic imaging for metabolic characterization of demyelinating plaques. Ann Neurol.

[97] Rooney WD, Goodkin DE, Schuff N, Meyerhoff DJ, Norman D, Weiner MW (1997). 1H MRSI of normal appearing white matter in multiple sclerosis. Mult Scler.

[98] Tedeschi G, Bonavita S, Mcfarland HF, Richert N, Duyn JH, Frank JA (2002). Proton MR spectroscopic imaging in multiple sclerosis. Neuroradiology.

[99] Dominique Sappey-Marinier, Matthieu Bagory, Salem Hannoun, Danielle, Ibarrola1, Confavreux C, Durand-Dubief F. Characterization of neurodegenerative processes in multiple sclerosis using magnetic resonance spectroscopic imaging and diffusion tensor imaging. MIAMS. 2008;60–70

[100] Husted CA, Goodin DS, Hugg JW, Maudsley AA, Tsuruda JS, de Bie SH, Fein G (1994). Biochemical alterations in multiple sclerosis lesions and normal-appearing white matter detected by in vivo 31P and 1H spectroscopic imaging. Ann Neurol.

[101] Inglese M, Liu S, Babb JS, Mannon LJ, Grossman RI, Gonen O (2004). Three-dimensional proton spectroscopy of deep gray matter nuclei in relapsing-remitting MS. Neurology.

[102] Kirov II, Tal A, Babb JS, Herbert J, Gonen O (2013). Serial proton MR spectroscopy of gray and white matter in relapsing-remitting MS. Neurology.

[103] Chang L, Munsaka SM, Kraft-Terry S, Ernst T (2013). Magnetic resonance spectroscopy to assess neuroinflammation and neuropathic pain. J Neuroimmune Pharmacol.

[104] Suhy J, Rooney WD, Goodkin DE, Capizzano AA, Soher BJ, Maudsley AA, Waubant E (2000). 1H MRSI comparison of white matter and lesions in primary progressive and relapsing-remitting MS. Mult Scler.

[105] Chard DT, Griffin CM, Mclean MA, Kapeller P, Kapoor R, Thompson AJ, Miller DH (2002). Brain metabolite changes in cortical grey and normal-appearing white matter in clinically early relapsing-remitting multiple sclerosis. Brain.

[106] Sharma R, Narayana PA, Wolinsky JS (2001). Grey matter abnormalities in multiple sclerosis: proton magnetic resonance spectroscopic imaging. Mult Scler.

[107] Tiberio M, Chard DT, Altmann DR, Davies G, Griffin CM, Mclean MA, Rashid W (2006). Metabolite changes in early relapsing-remitting multiple sclerosis. A 2 year follow-up study. J Neurol.

[108] Ratiney H, Okuda D, Graveron-Demilly D, Nelson SJ, Hauser S, Pelletier D. Estimation of myo-inositol and macromolecule contents in normal-appearing white and gray matter in MS using 3D-HMRSI at 3T. Proc Intl Soc Mag Reson Med. 2006;14:2635.

[109] Aboul-Enein F, Krssak M, Hoftberger R, Prayer D, Kristoferitsch W (2010). Reduced NAA-levels in the NAWM of patients with MS is a feature of progression. A study with quantitative magnetic resonance spectroscopy at 3 Tesla. Plos One.

[110] Khan O, Seraji-Bozorgzad N, Bao F, Razmjou S, Caon C, Santiago C, Latif Z, et al. The relationship between brain MR spectroscopy and disability in multiple sclerosis: 20-year data from the U.S. Glatiramer acetate extension study. J Neuroimaging. 2017;27(1):97–106. 10.1111/jon.12358 PMC5248608 27214389

[111] Kapeller P, Mclean MA, Griffin CM, Chard D, Parker GJ, Barker GJ, Thompson AJ (2001). Preliminary evidence for neuronal damage in cortical grey matter and normal appearing white matter in short duration relapsing-remitting multiple sclerosis: a quantitative MR spectroscopic imaging study. J Neurol.

[112] Sijens PE, Irwan R, Potze JH, Mostert JP, De Keyser J, Oudkerk M (2005). Analysis of the human brain in primary progressive multiple sclerosis with mapping of the spatial distributions using 1H MR spectroscopy and diffusion tensor imaging. Eur Radiol.

[113] Rahimian N, Saligheh Rad H, Firouznia K, Ebrahimzadeh SA, Meysamie A, Vafaiean H, Harirchian MH (2013). Magnetic resonance spectroscopic findings of chronic lesions in two subtypes of multiple sclerosis: primary progressive versus relapsing remitting. Iran J Radiol.

[114] Pelletier D, Nelson SJ, Grenier D, Lu Y, Genain C, Goodkin DE (2002). 3-D echo planar (1) HMRS imaging in MS: metabolite comparison from supratentorial vs. central brain. Magn Reson Imaging.

[115] Mathiesen HK, Jonsson A, Tscherning T, Hanson LG, Andresen J, Blinkenberg M, Paulson OB (2006). Correlation of global N-acetyl aspartate with cognitive impairment in multiple sclerosis. Arch Neurol.

[116] De Stefano N, Filippi M (2007). MR spectroscopy in multiple sclerosis. J Neuroimaging.

[117] Donadieu M, Le Fur Y, Lecocq A, Maudsley AA, Gherib S, Soulier E, Confort-Gouny S, et al. Metabolic voxel-based analysis of the complete human brain using fast 3D-MRSI: proof of concept in multiple sclerosis. J Magn Reson Imaging. 2016;44(2):411–9. 10.1002/jmri.25139 PMC4940345 26756662

[118] Mostert JP, Blaauw Y, Koch MW, Kuiper AJ, Hoogduin JM, De Keyser J (2008). Reproducibility over a 1-month period of 1H-MR spectroscopic imaging NAA/Cr ratios in clinically stable multiple sclerosis patients. Eur Radiol.

[119] Vafaeyan H, Ebrahimzadeh SA, Rahimian N, Alavijeh SK, Madadi A, Faeghi F, Harirchian MH (2015). Quantification of diagnostic biomarkers to detect multiple sclerosis lesions employing (1) H-MRSI at 3 T. Australas Phys Eng Sci Med.

